# Astrocytic FDX1 Contributes to Copper Dyshomeostasis‐associated Synaptic Dysfunction in Depression and Is Modulated by Exercise

**DOI:** 10.1002/advs.76088

**Published:** 2026-06-15

**Authors:** Lina Gao, Rongji Hui, Zhibo Tang, Tao Feng, Zhihang Hu, Xiaoqing Zhang, Ping Jiang, Hui Zhao, Kwok‐Fai So, Tianyuan Luo, Yanzhou Chang, Lan Yan

**Affiliations:** ^1^ School of Mental Health Wenzhou Medical University Wenzhou China; ^2^ College of Forensic Medicine Hebei Key Laboratory of Forensic Medicine Collaborative Innovation Center of Forensic Medical Molecular Identification Hebei Medical University Shijiazhuang China; ^3^ Hebei Medical University Basic Medicine Postdoctoral Research Station Shijiazhuang China; ^4^ Shanghai Mental Health Center School of Medicine Shanghai Jiao Tong University Shanghai China; ^5^ Liver Transplantation Center Department of General Surgery Huashan Hospital Fudan University Shanghai China; ^6^ Department of Psychiatry Ganzhou Hospital‐Nanfang Hospital Southern Medical University (Ganzhou People's Hospital) Ganzhou China; ^7^ GHM Institute of CNS Regeneration Jinan University Guangzhou China; ^8^ Department of Anesthesiology Affiliated Hospital of Zunyi Medical University Zunyi China; ^9^ Key Laboratory of Anesthesia and Organ Protection of Ministry of Education (In Cultivation) Zunyi Medical University Zunyi China; ^10^ College of Chemistry and Materials Science Jinan University Guangzhou China

**Keywords:** astrocytes, copper metabolism, depression, exercise, prelimbic cortex

## Abstract

Major depressive disorder (MDD) is increasingly linked to astrocyte dysfunction, yet how systemic metabolic disturbances contribute to glial alterations remains unclear. Disruption of trace metal homeostasis, particularly copper, has emerged as a potential contributor, but the underlying cellular mechanisms are poorly defined. Here, we integrate clinical data and mouse models to investigate the role of copper dyshomeostasis in depression. We show that copper levels are elevated in the systemic circulation of patients with MDD and in the prelimbic cortex (PrL) of stressed mice. In mice, copper accumulation is associated with increased astrocytic ferredoxin 1 (FDX1) expression, accompanied by reduced astrocyte number and structural complexity, impaired calcium signaling, and disrupted excitatory synaptic function. Astrocyte‐specific manipulations, in vivo calcium imaging, and electrophysiological recordings demonstrate that astrocytic FDX1 mediates the effects of copper imbalance on neural circuit dysfunction. Notably, physical exercise restores copper homeostasis, normalizes astrocytic FDX1 expression, improves astrocyte–neuron coupling, and alleviates depressive‐like behaviors. These findings identify an astrocyte‐mediated mechanism through which systemic copper imbalance influences neural function and provide insight into how exercise may mitigate depression‐related neural dysfunction.

## Graphical abstract

Chronic stress disrupts copper homeostasis and promotes copper accumulation in the prelimbic cortex, leading to astrocytic FDX1 upregulation. Elevated astrocytic FDX1 impairs calcium signaling, induces structural atrophy, and disrupts synaptic function, contributing to depressive‐like behaviors. Physical exercise reverses these alterations by restoring copper balance and improving astrocyte–neuron coupling.

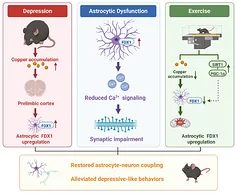

## 1 Introduction

Major depressive disorder (MDD) is a highly prevalent psychiatric disorder characterized by persistent low mood, anhedonia, and cognitive impairment, imposing a substantial burden on individuals and society. Epidemiological studies estimate that depression affects over 300 million individuals globally and constitutes a leading cause of disability worldwide [1, 2]. Despite extensive research, the pathophysiological mechanisms underlying MDD remain incompletely understood, and current therapeutic strategies are often limited in efficacy [3, 4]. Increasing evidence suggests that, in addition to classical neurotransmitter imbalance, glial cell dysfunction plays an essential role in the development of depression [5, 6].

Astrocytes are key cellular components of the central nervous system (CNS) and are increasingly recognized as important regulators of neuroimmune‐related processes [7, 8]. These cells maintain synaptic function, regulate neurotransmitter cycling, and modulate neuronal activity through calcium signaling. Under pathological conditions, astrocytes can undergo functional and structural alterations, including changes in morphology and calcium activity, which may contribute to synaptic dysfunction and behavioral abnormalities. Emerging studies have highlighted that astrocyte dysfunction is closely associated with depression, particularly in brain regions such as the prelimbic cortex (PrL), which is critically involved in emotional regulation [9, 10]. Together, these findings underscore the importance of astrocytes in maintaining neural circuit stability in mood‐related brain regions.

Intriguingly, emerging evidence points to disruptions in trace metal homeostasis, particularly copper, as potential contributors to such astrocytic and synaptic dysfunction in the CNS. Among these metals, copper plays a pivotal role in mitochondrial respiration, antioxidant defense, and enzymatic reactions [11, 12, 13, 14, 15, 16]. Disruption of copper homeostasis has been increasingly implicated in CNS dysfunction and may influence glial cell function [17, 18]. Clinical studies have reported elevated serum copper levels in patients with MDD, suggesting a potential association between copper dysregulation and depressive pathology [19, 20]. These observations raise the possibility that copper imbalance may affect neural circuit function through cell‐type‐specific mechanisms. Ferredoxin 1 (FDX1), a mitochondrial protein involved in electron transfer and cellular metabolism, has recently been identified as an important regulator of copper‐dependent processes, including mitochondrial protein lipoylation and copper‐induced metabolic stress [21]. Given its role in copper metabolism, FDX1 may represent a potential molecular link between copper dyshomeostasis and astrocyte dysfunction. Whether this copper–FDX1 axis operates in astrocytes within depression‐related brain regions remains to be clarified.

Physical exercise is widely recognized as an effective non‐pharmacological intervention for depression. It has been shown to improve mood, enhance synaptic plasticity, and regulate neurobiological processes. Recent studies also suggest that exercise can modulate glial cell function and improve brain homeostasis [22, 23]. These findings suggest that exercise may engage glia‐related mechanisms to restore neural function, although the underlying molecular pathways remain incompletely understood and may involve multiple convergent processes.

In this study, we demonstrate that copper levels are significantly elevated in both the systemic circulation of patients with MDD and the prelimbic cortex of mice subjected to chronic stress. We further show that copper accumulation is associated with increased expression of FDX1 in astrocytes. Mechanistically, aberrant astrocytic FDX1 leads to structural simplification and impaired calcium signaling in astrocytes, which in turn disrupts neuronal synaptic function. These findings indicate that astrocytes serve as an important cellular target linking copper dyshomeostasis to neural circuit dysfunction. Furthermore, exercise effectively reverses these alterations, in part by restoring copper homeostasis and regulating astrocytic FDX1 signaling. Collectively, our results identify an astrocyte‐mediated mechanism linking copper dyshomeostasis to synaptic dysfunction and provide new insight into glial contributions to depression.

## 2 Results

### 2.1 Physical Exercise Attenuates Elevated Systemic Copper levels Associated with Depression

**FIGURE 1:**
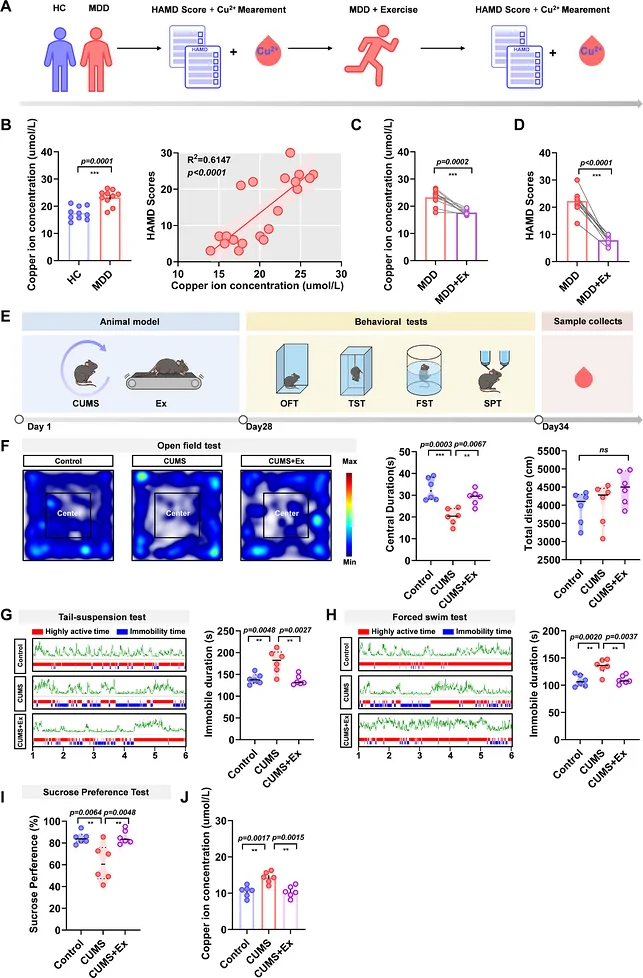
Exercise reduces elevated copper levels and ameliorates depressive‐like behaviors in patients with MDD and in CUMS mice. (A) Schematic of the human cohort study design. Healthy controls and MDD patients were enrolled for HAMD assessment and serum copper measurement. (B) Left: Serum copper levels were higher in MDD patients compared to controls (unpaired two‐tailed *t*‐test, t (18)  =  4.891, P  =  0.0001; n  =  10/group). Right: Serum copper positively correlated with HAMD scores in MDD patients (simple linear regression, R^2 ^ =  0.6147, P < 0.0001; n  =  10). (C) Exercise lowered serum copper in MDD patients (paired two‐tailed t‐test, MDD vs. MDD+Ex: t(9)  =  6.173, P  =  0.0002; n  =  10). (D) Exercise reduced HAMD scores in MDD patients (paired two‐tailed t‐test, MDD vs. MDD+Ex: t(9)  =  11.87, P < 0.0001; n  =  10). (E) Experimental timeline of the CUMS mouse model, including a subgroup with treadmill exercise (Ex). (F) Left: Representative open‐field heatmaps. Right: Center time (one‐way ANOVA, F(2,15)  =  14.36, P  =  0.0003) and total distance (F(2,15)  =  2.010, P  =  0.1685; n  =  6/group). (G) Left: Representative TST activity traces. Right: Immobility time in TST (one‐way ANOVA, F(2,15)  =  10.32, P  =  0.0015; n  =  6/group). (H) Left: Representative FST activity traces. Right: Immobility time in FST (one‐way ANOVA, F(2,15)  =  11.05, P  =  0.0011; n  =  6/group). (I) Sucrose preference index (one‐way ANOVA, F(2,15)  =  9.201, P  =  0.0025; n  =  6/group). (J) Serum copper levels in mice (one‐way ANOVA, F(2,15)  =  12.62, P  =  0.0006; n  =  6/group).

A population‐based study was first conducted to assess the role of copper in depression. Patients with MDD showed significantly higher serum copper levels than controls, and these levels were positively correlated with HAMD scores (Figure 1A,B), suggesting that perturbed copper metabolism may be closely linked to the clinical manifestation of depression. Building upon these findings, MDD patients underwent a one‐month regimen of structured physical exercise. Post‐intervention analyses demonstrated a marked reduction in serum copper levels, concomitant with significant improvements in HAMD scores (Figure 1C,D). These observations indicate that physical exercise can effectively normalize copper metabolism associated with depressive states.

To explore these findings experimentally, the CUMS mouse model was used (Figure 1E). CUMS‐exposed mice exhibited depressive‐like behaviors, including reduced central zone time, prolonged immobility, and decreased sucrose preference (Figure 1F,I). Importantly, treadmill exercise intervention (10 m/min, 1 h/day) significantly ameliorated these behavioral deficits. Corroborating the behavioral findings, CUMS mice displayed elevated serum copper levels, which were effectively reversed by exercise training (Figure 1J). Meanwhile, exogenous copper supplementation induced depression‐like behaviors in mice, whereas administration of the copper chelator ammonium tetrathiomolybdate effectively suppressed these behaviors (Figure S1A–J). Collectively, these population‐ and animal‐based findings indicate that elevated copper levels represent a characteristic feature of depressive states, and that physical exercise acts as a modulator of copper homeostasis, contributing to its antidepressant effects.

### 2.2 Exercise Restores Copper Homeostasis in the PrL and Alleviates Depressive‐Like Behaviors

**FIGURE 2:**
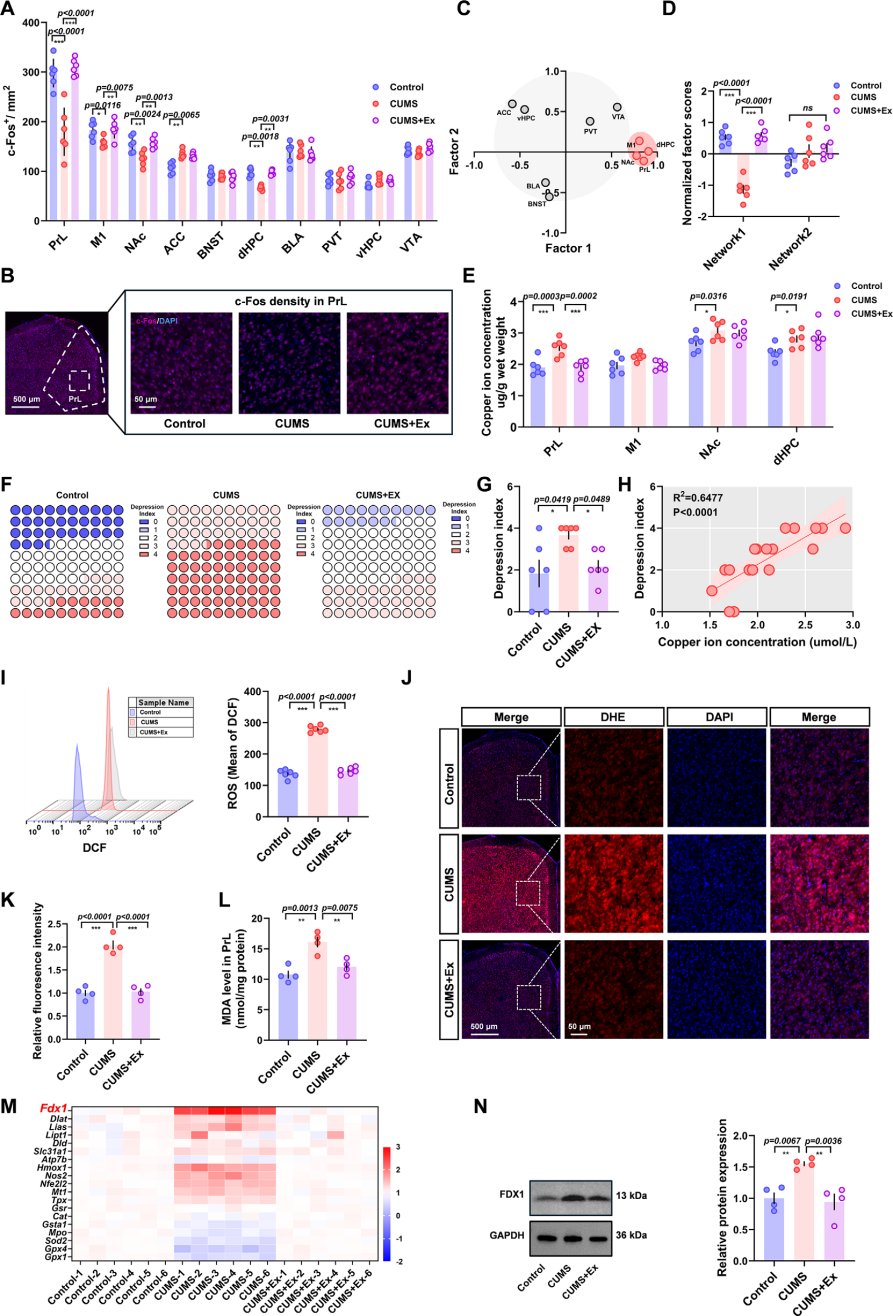
CUMS‐induced brain region‐specific copper dyshomeostasis and oxidative stress, which are reversed by exercise intervention. (A) Quantification of c‐Fos^+^ cell density in multiple depression‐related regions: PrL, M1, NAc, ACC, BNST, dHPC, BLA, PVT, vHPC, and VTA (two‐way ANOVA, F(18,150)  =  13.89, P < 0.0001; n  =  6/group) (B) Representative immunofluorescence images of c‐Fos in PrL. (C) Factor analysis of c‐Fos activity identified two functional networks: Network 1 (PrL, M1, NAc, dHPC) and Network 2 (ACC, BNST, BLA, PVT, vHPC, VTA). (D) Activity of Network 1 was reduced in CUMS mice and restored by exercise; Network 2 was unchanged (two‐way ANOVA, F(2,30)  =  28.53, P < 0.0001; n  =  6/group). (E) Copper concentrations in Network 1 regions (PrL, M1, NAc, dHPC) (two‐way ANOVA, F(6,60) = 2.755, p = 0.0197; n  =  6/group). (F) A composite depression index was calculated from OFT, TST, FST, and SPT performances (1 point assigned for each parameter beyond control‐group thresholds). (G) Depression index across groups (Kruskal‐Wallis test, H = 5.049, P  =  0.0211) (H) PrL copper levels positively correlated with the depression index (simple linear regression, R^2^  =  0.6477, P < 0.0001; n  =  6/group). (I) Flow‐cytometric quantification of DCFH‐DA fluorescence in brain tissue (one‐way ANOVA, F(2,15)  =  279.6, P < 0.0001; n  =  6/group). (J, K) Representative DHE staining in PrL (J) and quantified fluorescence (K) (one‐way ANOVA, F(2,9)  =  54.14, P < 0.0001; n  =  4/group). (L) MDA levels in PrL (one‐way ANOVA, F(2,9)  =  15.30, P  =  0.0013; n  =  4/group). (M) Heatmap showing qPCR‐based expression of copper/oxidative‐stress‐related genes (including FDX1) in PrL. (N) Representative immunoblot and quantification of FDX1 protein in PrL (one‐way ANOVA, F(2,9)  =  12.58, P  =  0.0025; n  =  4/group).

To investigate central mechanisms, this study first systematically screened functional brain regions associated with depressive states and exercise interventions through comprehensive whole‐brain mapping analysis of the immediate early gene c‐Fos [24]. Among multiple emotion‐related regions, including the PrL, primary motor cortex (M1), nucleus accumbens (NAc), anterior cingulate cortex (ACC), and dorsal hippocampus (dHPC), the PrL in CUMS‐exposed mice exhibited the most pronounced reduction of c‐Fos expression. Notably, treadmill exercise intervention effectively reversed the suppression of neuronal activity in the PrL induced by chronic stress (Figure 2A,B; Figure S2A). Factor analysis of the c‐Fos data revealed that Network 1, comprising the PrL, M1, NAc, and dHPC, accounted for 44.93% of the total variance in activity, whereas Network 2, including the ACC, bed nucleus of the stria terminalis (BNST), basolateral amygdala (BLA), paraventricular thalamus (PVT), ventral hippocampus (vHPC), and ventral tegmental area (VTA), accounted for 17.58% (Figure 2C,D). Importantly, the activity of Network 1 brain regions decreased in the CUMS group and increased in the exercise group. Subsequently, we quantified copper concentrations in the four brain regions encompassed within Network 1. The results demonstrated that CUMS induced a significant elevation of copper in the PrL, NAc, and dHPC. Notably, exercise intervention restored copper levels toward control levels in the PrL (Figure 2E). To quantify overall depressive‐like behavioral changes, a composite “depression index” was constructed by combining multiple behavioral tests based on a previously published method [25]. Analysis revealed that CUMS exposure markedly increased the depression index, whereas exercise intervention significantly reduced it (Figure 2F,G). Critically, copper levels in the PrL exhibited a strong positive correlation with the depression index (Figure 2H), identifying disrupted PrL copper homeostasis as an important substrate for depressive behaviors and a target for the antidepressant effects of exercise.

Although elevated copper levels in the PrL are closely associated with depressive‐like behaviors, the downstream molecular and cellular mechanisms remain incompletely understood. Previous studies indicate that copper homeostasis plays a critical role in maintaining mitochondrial redox balance [26]. Consistent with these findings, we observed that CUMS markedly increased ROS and MDA levels in the PrL, both of which were significantly attenuated by exercise intervention (Figure 2I–L). Quantitative PCR analyses showed that CUMS significantly disrupted transcription of multiple genes involved in copper homeostasis and redox regulation. Among these, *Fdx1* exhibited a particularly robust upregulation, which was effectively reversed by exercise intervention (Figure 2M). In parallel, the expression of genes involved in antioxidant defense was broadly suppressed, indicating an impaired antioxidative capacity in the PrL under depressive conditions and a heightened susceptibility to copper‐dependent oxidative stress. Consistent with these transcriptional changes, protein‐level analyses further confirmed a marked increase in FDX1 expression in the PrL following CUMS exposure, which was significantly reduced by exercise intervention, thereby corroborating the gene expression findings (Figure 2N). These results suggest that stress‐induced copper dysregulation in the PrL enhances oxidative stress and aberrantly upregulates FDX1 expression, whereas exercise mitigates local oxidative stress and suppresses pathological FDX1 upregulation, which may contribute to the antidepressant effects of exercise.

### 2.3 Astrocytes are a Major Cellular Target of FDX1 Regulation by Exercise in the PrL

**FIGURE 3:**
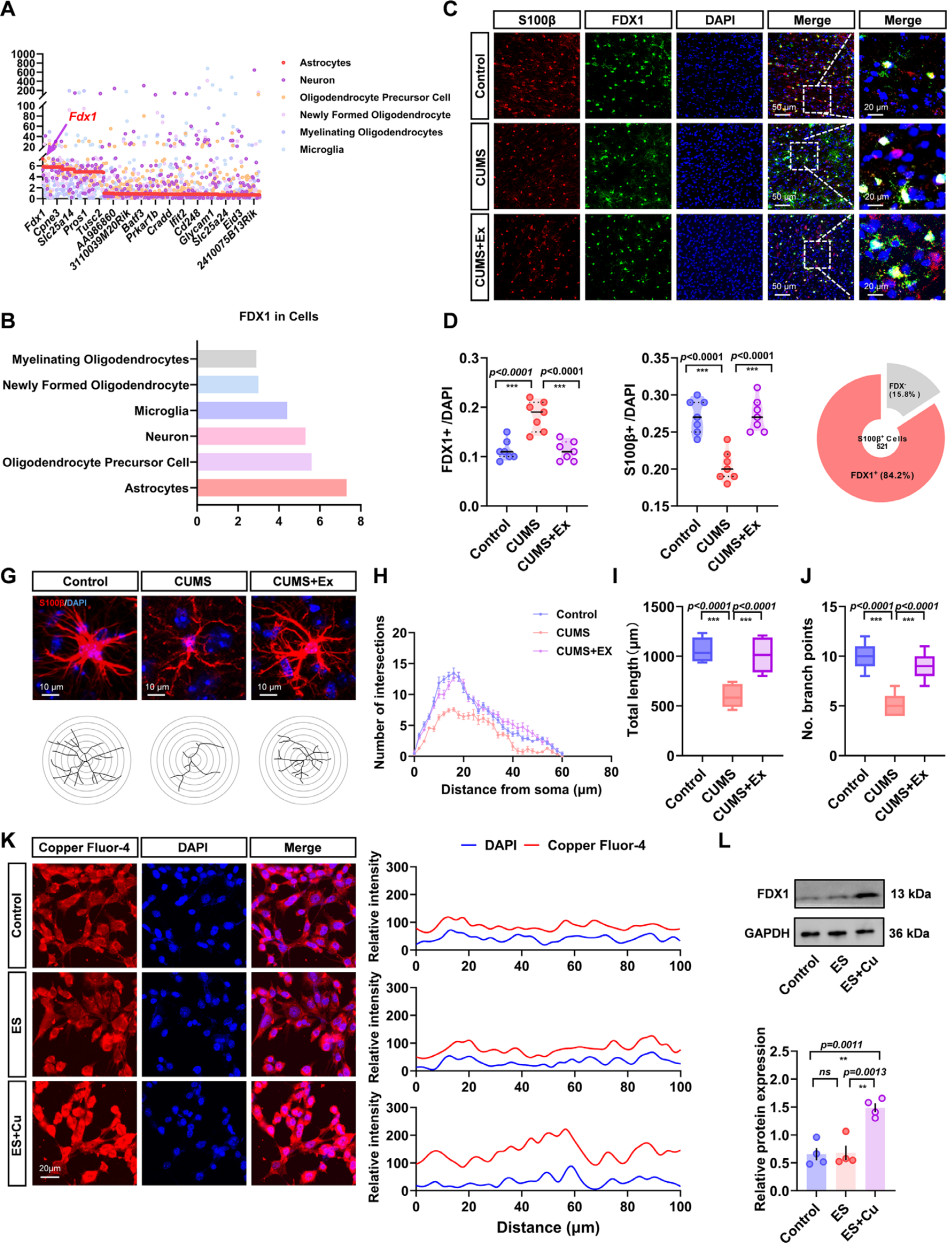
FDX1 is enriched in astrocytes, and its upregulation is associated with astrocytic structural alterations following CUMS. (A) Relative expression of FDX1 and selected genes across major CNS cell types. (B) FDX1 expression across brain cell types, showing enrichment in astrocytes. (C) Representative immunofluorescence images of FDX1 colocalization with astrocytic marker S100β in the PrL. (D, E) Quantification of FDX1^+^ cells (D) and S100β^+^ cells (E). Exercise reversed the CUMS‐induced increase in FDX1^+^ cells and decrease in S100β^+^ cells (one‐way ANOVA, F(2, 18) = 20.26, P < 0.0001 for D; F(2, 18) = 22.50, P < 0.0001 for E; n  =  7/group). (F) Proportion of FDX1‐expressing cells within the S100β^+^ astrocyte population. (G) Representative astrocyte images and corresponding Sholl‐analysis skeletons. (H–J) Sholl analysis: intersections vs. distance from soma (H), total process length (I), and branch number (J). CUMS induced astrocytic structural atrophy and reduced complexity, which were rescued by exercise (one‐way ANOVA, I: F(2,18)  =  27.02, P < 0.0001; J: F(2,18)  =  28.72, P < 0.0001; n  =  7/group). (K) Representative live‐cell confocal images of primary astrocytes stained with Copper Fluor‐4 for detection of intracellular Cu^+^ levels. Quantitative line‐scan analysis of fluorescence intensity profiles is shown on the right. (L) Representative western blot images and quantification of FDX1 protein expression in primary astrocytes (one‐way ANOVA, F(2,9) = 19.16, p = 0.0006, n  =  4/group).

To identify the cellular source of FDX1, we analyzed PrL single‐cell data. FDX1 was broadly expressed, with the highest expression levels in astrocytes (Figure 3A,B). Immunofluorescence confirmed that ∼84.2% of S100β^+^ astrocytes co‐expressed FDX1. CUMS increased FDX1^+^ cell density, which exercise normalized (Figure 3C,D). CUMS also reduced S100β^+^ astrocyte density, restored by exercise (Figure 3E,F). About 22.6% of NeuN^+^ neurons were FDX1^+^, but neuronal density was unaffected by CUMS (Figure S3A–D). Minimal FDX1/Olig2 co‐localization indicated oligodendrocytes are not the main source (Figure S3E–H).

We next assessed whether aberrant FDX1 expression was associated with astrocytic structural and functional alterations. Sholl analysis revealed that CUMS significantly reduced astrocytic process number, total process length, and branching complexity, indicating impairments in structural integrity and functional capacity. Notably, exercise intervention substantially mitigated these morphological deficits (Figure 3G–J). To test whether elevated copper could directly drive FDX1 upregulation in astrocytes, we treated cultured primary astrocytes with copper chloride (CuCl_2_) together with elesclomol (ES), a compound that facilitates copper uptake into cells. Compared with vehicle‐treated or ES‐only controls, copper supplementation significantly increased FDX1 expression (Figure 3K,L). We then asked whether astrocytic FDX1 plays a causal role in copper‐induced depressive‐like behaviors. To address this, we selectively knocked down FDX1 in PrL astrocytes using viral‐mediated gene silencing before exposing mice to exogenous copper. This astrocyte‐specific knockdown significantly attenuated copper‐induced depressive‐like behaviors (Figure S4A–H).

Having shown that astrocytic FDX1 contributes to copper‐induced depressive‐like behaviors, we next explored the underlying molecular mechanism. FDX1 has been implicated in the regulation of protein lipoylation, particularly lipoylated DLAT (Lip‐DLAT). We therefore asked whether copper‐induced FDX1 upregulation was accompanied by Lip‐DLAT accumulation. In cultured astrocytes, copper supplementation markedly increased Lip‐DLAT levels (Figure S4I). Importantly, the same alteration was observed in vivo. CUMS‐exposed mice showed markedly elevated Lip‐DLAT levels in the PrL, and this effect was reversed by exercise (Figure S4J). Taken together, CUMS exposure in the PrL is characterized by simultaneous increases in FDX1^+^ cell number and decreases in astrocyte density and structural complexity, whereas exercise reverses these changes. The concordance of these alterations suggests that abnormal upregulation of FDX1 may be closely associated with compromised astrocytic integrity.

### 2.4 Exercise Regulates ASTROCYTIC FDX1 in the PrL and Contributes to its Antidepressant Effects

**FIGURE 4:**
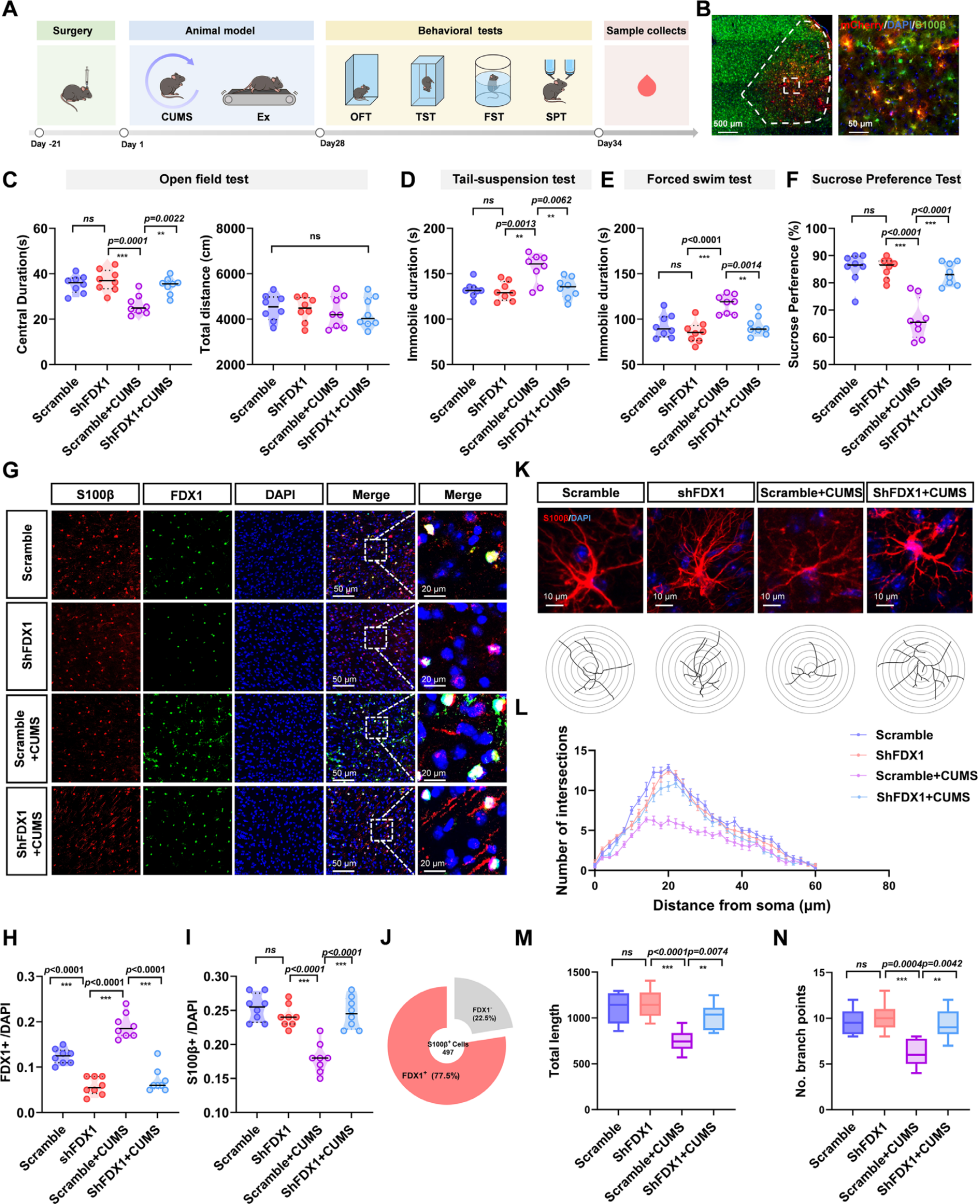
Astrocyte‐specific knockdown of FDX1 in the PrL alleviates CUMS‐induced depressive‐like behaviors and astrocytic structural deficits. (A) Experimental timeline of viral‐mediated FDX1 knockdown and behavioral testing. Mice received stereotaxic injection of rAAV‐GfaABC1D‐mCherry‐5'‐miR‐30a‐shRNA(Fdx1)‐3'‐miR‐30a‐WPRE or control virus rAAV‐GfaABC1D‐mCherry‐5'‐miR‐30a‐shRNA(scramble)‐3'‐miR‐30a‐WPRE. (B) Representative images of viral injection sites in the PrL. (C–F) Behavioral tests showing FDX1 knockdown alleviated CUMS‐induced depressive‐like behaviors: Open field (center time): one‐way ANOVA, F(3,28)  =  10.38, P < 0.0001; total distance: F(3,28)  =  0.2362, P  =  0.8703. Tail suspension: F(3,28)  =  7.811, P  =  0.0006. Forced swim: F(3,28)  =  11.15, P < 0.0001. Sucrose preference: F(3,28)  =  22.31, P < 0.0001; n  =  8/group. (G) Representative immunofluorescence of FDX1 in S100β^+^ astrocytes in PrL. (H, I) Quantification of FDX1^+^ cells (H) and S100β^+^ cells (I) (one‐way ANOVA: H, F(3, 28) = 54.14, P < 0.0001; I, F(3, 28) = 22.12, P < 0.0001; n  =  8/group). (J) Proportion of FDX1‐expressing cells within S100β^+^ astrocytes. (K) Representative high‐magnification astrocyte images and Sholl skeletons. (L–N) Sholl analysis: intersections vs. distance (L), total process length (M), and branch number (N). FDX1 knockdown reversed CUMS‐induced astrocytic atrophy and complexity loss (one‐way ANOVA: M, F(3,28)  =  11.83, P < 0.0001; N, F(3,28)  =  11.67, P < 0.0001; n  =  8/group).

Having identified astrocytes as a major cellular target associated with the antidepressant effects of exercise, we proceeded to examine whether FDX1 in astrocytes modulates depressive‐like behaviors. To this end, we selectively knocked down FDX1 in PrL astrocytes via viral‐mediated gene silencing (Figure 4A,B). The efficacy of FDX1 knockdown in PrL astrocytes was confirmed (Figure S5A–C). Behavioral analyses revealed that astrocyte‐specific FDX1 knockdown significantly alleviated depressive‐like phenotypes induced by CUMS (Figure 4C–F). In terms of number, chronic stress led to a reduction in astrocyte number, whereas FDX1 knockdown rescued the stress‐induced decrease in astrocyte number (Figure 4G–J). At the cellular morphological level, Sholl analysis demonstrated that CUMS markedly reduced astrocytic process number, total process length, and branching complexity in the PrL, which was effectively rescued by FDX1 knockdown (Figure 4K–N). Furthermore, astrocyte‐specific FDX1 knockdown suppressed the aberrant elevation of Lip‐DLAT and ameliorated CUMS‐induced increases in ROS and MDA levels (Figure S5D–G). Notably, knockdown of FDX1 in PrL neurons did not significantly alter depressive‐like behaviors (Figure S6A–M), further underscoring the astrocyte‐specific role of FDX1 in modulating stress‐induced behavioral deficits.

**FIGURE 5:**
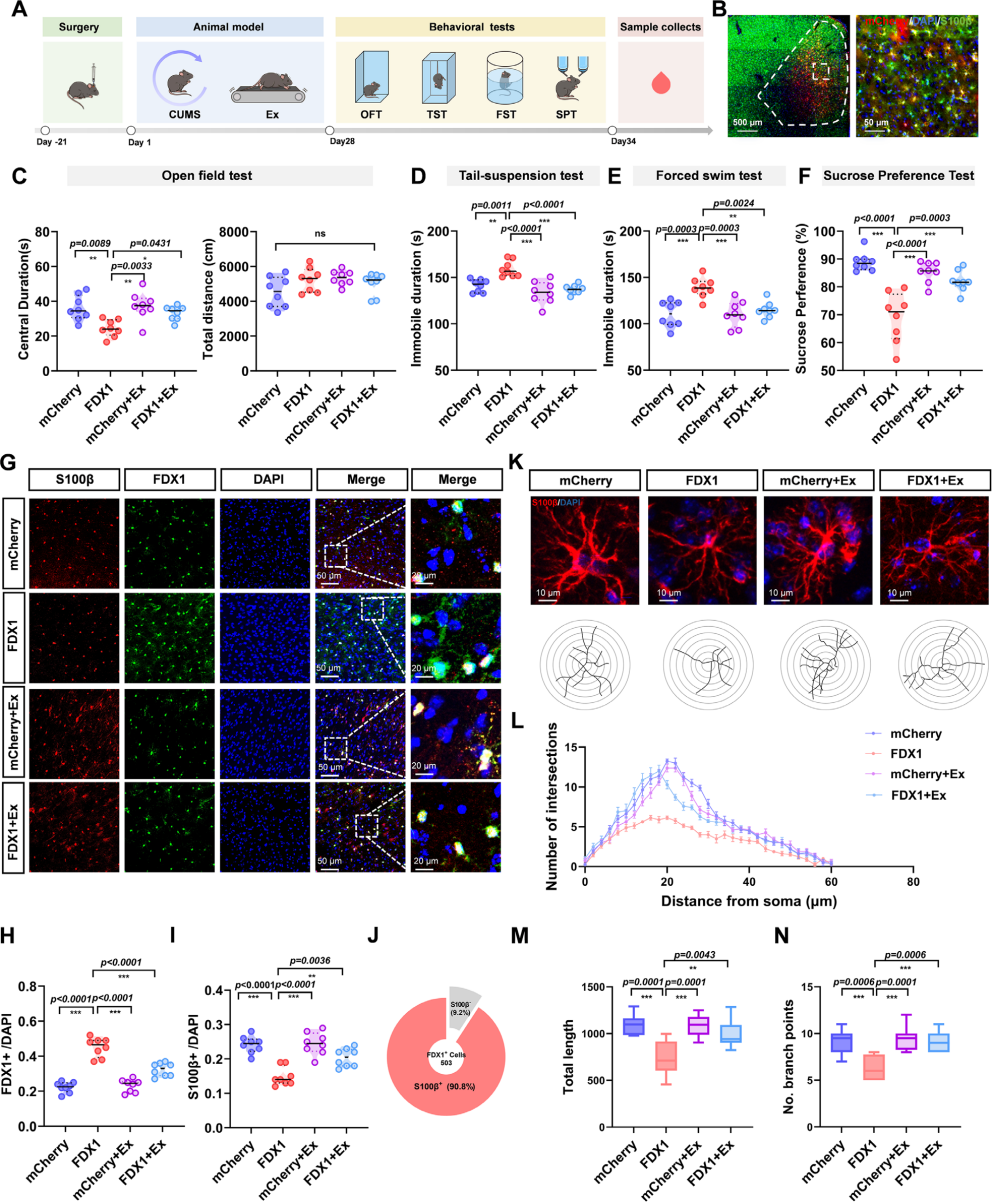
Overexpression of FDX1 in the PrL induces depressive‐like behaviors and astrocytic structural abnormalities, which are ameliorated by exercise. (A) Timeline of viral manipulation and exercise intervention. Mice received stereotaxic injection of rAAV‐GfaABC1D‐Fdx1‐P2A‐mCherry‐WPRE or control virus rAAV‐GfaABC1D‐mCherry‐WPRE. (B) Representative images of viral injection sites in PrL. (C–F) FDX1 overexpression induced depressive‐like behaviors, reversed by exercise: Open field center time: F(3,28)  =  6.906, P  =  0.0013; total distance: F(3,28)  =  2.305, P  =  0.0985. Tail suspension: F(3,28)  =  14.13, P < 0.0001. Forced swim: F(3,28)  =  10.37, P < 0.0001. Sucrose preference: F(3,28)  =  19.91, P < 0.0001. n  =  8/group. (G) Representative immunofluorescence of FDX1 in S100β^+^ astrocytes in PrL. (H, I) Quantification of FDX1^+^ and S100β^+^ cells in PrL (one‐way ANOVA: H, F(3, 28) = 54.53, P < 0.0001; I, F(3, 28) = 20.56, P < 0.0001; n  =  8/group). (J) Proportion of FDX1‐positive cells among S100β^+^ astrocytes. (K) High‐magnification astrocyte images and Sholl skeletons. (L–N) Sholl analysis: intersections vs. distance (L), total process length (M), branch number (N). FDX1 overexpression reduced astrocyte complexity, rescued by exercise (one‐way ANOVA: M, F(3,28)  =  11.69, P < 0.0001; N, F(3,28)  =  11.29, P < 0.0001; n  =  8/group).

To determine whether upregulation of FDX1 in astrocytes is sufficient to induce depressive‐like behaviors, we overexpressed FDX1 selectively in PrL astrocytes via viral delivery (Figure 5A,B). qPCR, Western blot, and immunofluorescence analyses confirmed robust astrocytic overexpression of FDX1 (Figure S7A–C). Overexpression induced pronounced depressive behaviors, including decreased center time, increased immobility, and reduced sucrose preference (Figure 5C–F), accompanied by a reduction in astrocyte number (Figure 5G–J), reduced astrocytic process complexity (Figure 5K–N), and aberrantly elevated Lip‐DLAT levels (Figure S7D). Furthermore, given that exercise also markedly ameliorated the depressive‐like behaviors induced by FDX1 overexpression, we investigated whether exercise may regulate FDX1 through pathways beyond copper regulation. Using qPCR, we examined several candidate signals that may influence FDX1 expression or its downstream effects. Among these, *Prkaa1*, *Sirt1*, and *Ppargc1a*, which encode key components associated with the SIRT1–PGC‐1α signaling pathway, were significantly increased after exercise (Figure S7E). Consistent with these transcriptional changes, protein‐level analyses further confirmed significant elevations of SIRT1 and PGC‐1α (Figure S7F). These results suggest that exercise may additionally engage SIRT1–PGC‐1α‐associated protective signaling under conditions of persistent FDX1 overexpression. Collectively, treadmill exercise reversed both the behavioral and structural deficits induced by FDX1 overexpression, indicating that modulation of astrocytic FDX1 is an important component of the antidepressant effects of exercise.

### 2.5 Aberrant Astrocytic FDX1 Impairs Neuronal Synaptic Plasticity and Contributes to Depressive‐Like Behaviors

**FIGURE 6:**
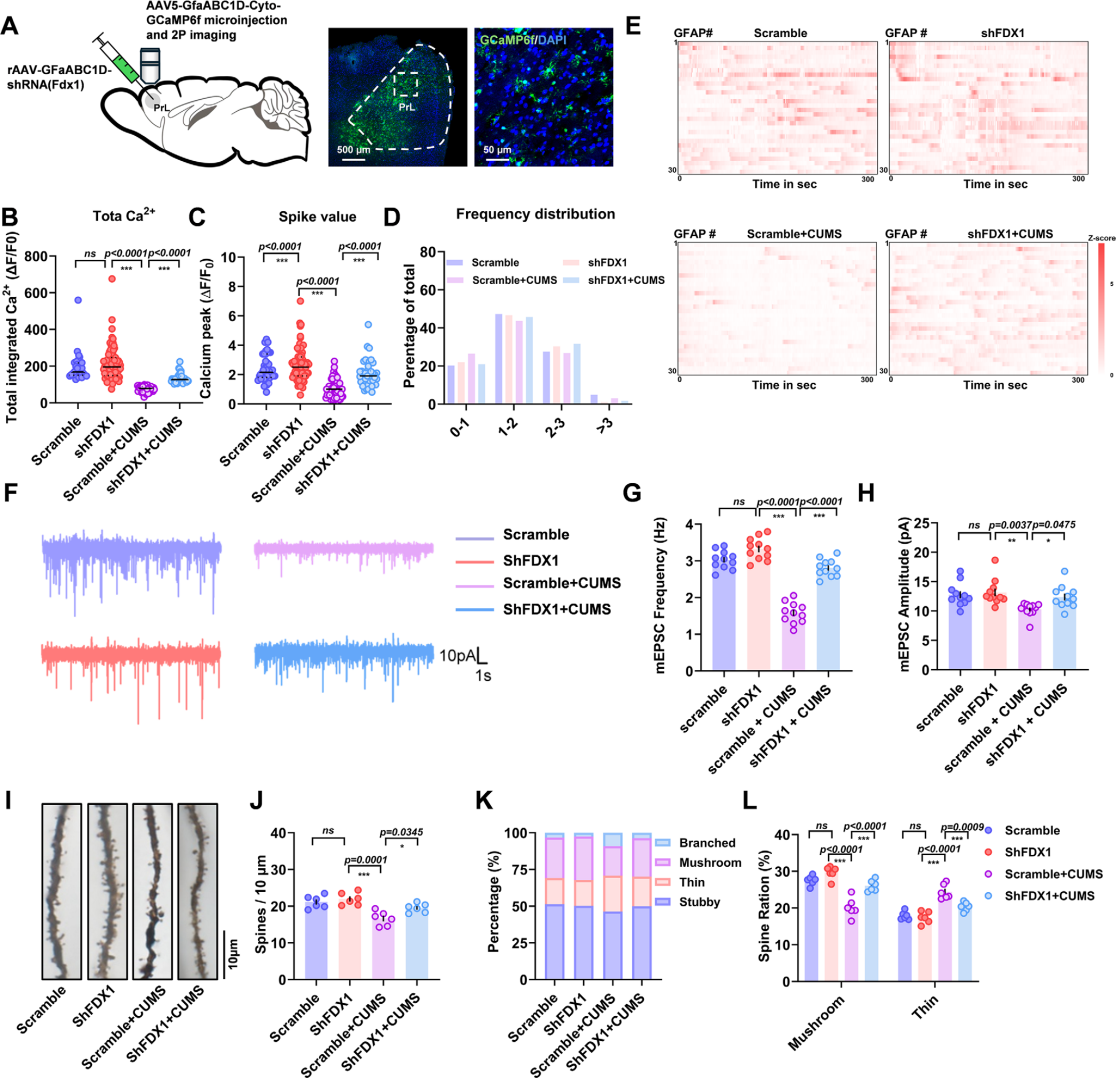
Knockdown of FDX1 restores astrocytic calcium signaling, synaptic transmission, and dendritic spine density in the PrL of CUMS mice. (A) Schematic and representative images of in vivo two‐photon Ca^2+^ imaging. Mice received stereotaxic injection of rAAV‐GfaABC1D‐cyto‐GCaMP6f‐WPRE together with either rAAV‐GFaABC1D‐mCherry‐5'miR‐30a‐shRNA(Fdx1)‐3'‐miR30a‐WPRE or control virus rAAV‐GFaABC1D‐mCherry‐5′miR‐30a‐shRNA(scramble)‐3′miR‐30a‐WPRE. (B) Integrated astrocytic Ca^2+^ signal reduced by CUMS, restored by FDX1 knockdown (Kruskal‐Wallis test, H = 138.4, P < 0.0001). (C) Single Ca^2+^ transient amplitude reduced by CUMS, rescued by FDX1 knockdown (Kruskal‐Wallis test, H = 80.73, P < 0.0001). (D) Distribution of Ca^2+^ transient frequency across groups. (E) Heat maps of normalized Ca^2+^ activity (Z‐score) over 300 s (30 cells from 5 mice). (F–H) mEPSC recordings in PrL neurons: representative traces (F), frequency (G), and amplitude (H) reduced by CUMS and restored by FDX1 knockdown (one‐way ANOVA: G, F(3,40)  =  88.28, P < 0.0001; H, F(3,40)  =  5.410, P  =  0.0032; n  =  11/group). (I) Representative dendritic segments from each group. (J) Dendritic spine density reduced by CUMS, rescued by FDX1 knockdown (one‐way ANOVA, F(3,20)  =  11.95, P  =  0.0001; n  =  6/group). (K) Spine‐subtype distribution: CUMS increased thin spines, decreased mushroom spines; FDX1 knockdown normalized proportions. (L) Quantification of mushroom and thin spine proportions (two‐way ANOVA, F(3,40)  =  55.00, P < 0.0001; n  =  6/group).

The complex morphology of astrocytes is fundamental to their calcium signaling function, and structural disruption can profoundly alter Ca^2+^ dynamics [27]. To examine whether aberrant expression of FDX1 in astrocytes influences calcium activity, we performed in vivo calcium imaging in PrL astrocytes. Exposure to CUMS markedly suppressed astrocytic Ca^2+^ activity, while astrocyte‐specific FDX1 knockdown effectively restored it. Importantly, CUMS and FDX1 knockdown altered the amplitude of individual calcium transients without affecting their frequency distribution, suggesting impaired calcium signaling strength during individual events (Figure 6A–E).

Astrocytic function is closely linked to neuronal activity. We tested whether astrocytic FDX1 upregulation affects synaptic plasticity via astrocytic Ca^2+^ signaling. Patch‐clamp recordings showed CUMS reduced mEPSC frequency and amplitude in PrL neurons, and astrocyte‐specific FDX1 knockdown reversed these deficits (Figure 6F–H). At the structural level, Golgi staining demonstrated that CUMS markedly reduced dendritic spine density in PrL neurons, particularly mature mushroom‐type spines. Astrocyte‐specific FDX1 knockdown mitigated these structural deficits, preserving dendritic spine density and morphology (Figure 6I–L).

**FIGURE 7:**
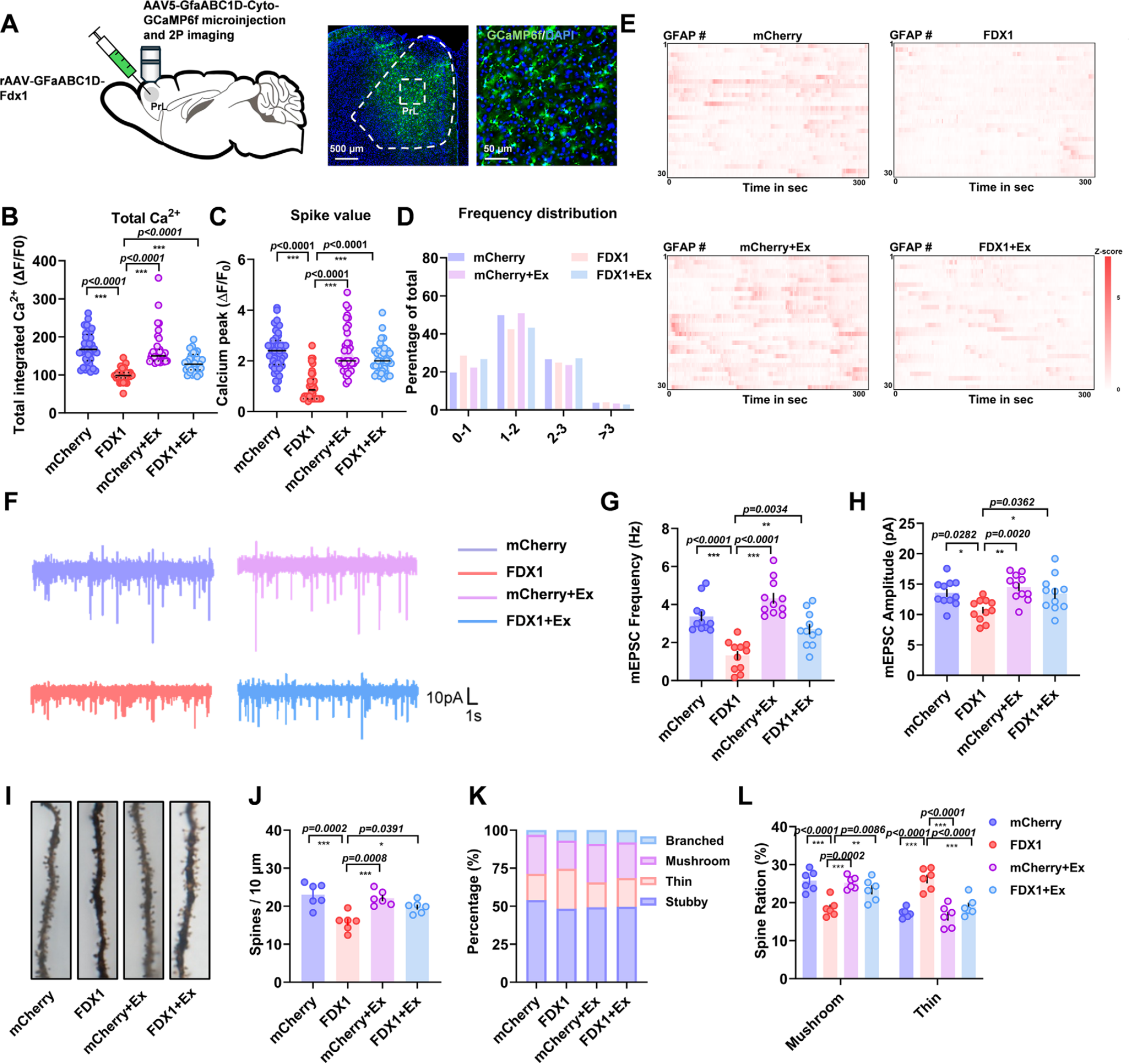
Astrocytic FDX1 regulates calcium signaling, synaptic transmission, and dendritic spine structure in the PrL, and these abnormalities are partially reversed by exercise. (A) Schematic and representative images of in vivo two‐photon Ca^2+^ imaging. Mice received stereotaxic injection of rAAV‐GfaABC1D‐cyto‐GCaMP6f‐WPRE together with either rAAV‐GfaABC1D‐Fdx1‐P2A‐mCherry‐WPRE or control virus rAAV‐GfaABC1D‐mCherry‐WPRE. (B) Integrated astrocytic Ca^2+^ signals decreased by FDX1 overexpression were rescued by exercise (Kruskal–Wallis test, H =  68.90, P < 0.0001). (C) Single Ca^2+^ transient amplitude decreased by FDX1 overexpression and restored by exercise (Kruskal–Wallis test, H = 56.12, P < 0.0001). (D) Ca^2+^ transient frequency was unaffected by FDX1 overexpression or exercise. (E) Heat maps of normalized Ca^2+^ activity (Z‐score) over 300 s (30 cells from 5 mice). (F–H) mEPSC recordings in PrL neurons: representative traces (F), frequency (G), and amplitude (H) were reduced by FDX1 overexpression and rescued by exercise (one‐way ANOVA: G, F(3,40)  =  22.97, P < 0.0001; H, F(3,40)  =  5.614, P  =  0.0026; n  =  11/group). (I) Representative dendritic segments from each group. (J) Dendritic spine density reduced by FDX1 overexpression was restored by exercise (one‐way ANOVA, F(3,20)  =  11.26, P  =  0.0002; n  =  6/group). (K) Spine subtype distribution: FDX1 overexpression increased thin spines and decreased mushroom spines; exercise normalized these proportions. (L) Quantification of mushroom and thin spine proportions (two‐way ANOVA, F(3,40)  =  30.00, P < 0.0001; n  =  6/group).

We further validated these findings by overexpressing FDX1 in PrL astrocytes. FDX1 overexpression significantly suppressed astrocytic Ca^2+^ activity, an effect that was restored by exercise intervention (Figure 7A–E). Correspondingly, mEPSC frequency and amplitude were markedly reduced in FDX1‐overexpressing conditions, and exercise effectively rescued these functional impairments (Figure 7F–H). Golgi staining further confirmed that FDX1 overexpression led to dendritic spine loss and a selective reduction in mushroom‐type spines, both of which were prevented by exercise (Figure 7I–L). Collectively, these results indicate that stress‐induced upregulation of astrocytic FDX1 decreases astrocytic Ca^2+^ influx, resulting in compromised excitatory synaptic function and impaired structural plasticity in neurons. Exercise reinstates functional astrocyte–neuron coupling by suppressing aberrant FDX1 expression, thereby ameliorating depressive‐like behaviors.

## 3 Discussion

Depression is a multifactorial psychiatric disorder involving complex interactions among neuronal, glial, and systemic factors. While classical hypotheses have primarily focused on neurotransmitter imbalance, increasing evidence highlights the critical role of glial cells, particularly astrocytes, in regulating neural circuit function and behavioral outcomes. In this context, understanding how systemic metabolic disturbances influence astrocyte function may provide important insight into the pathophysiology of depression.

The observation of elevated copper levels in both systemic circulation and the PrL aligns with prior clinical and preclinical studies indicating altered copper homeostasis in subsets of individuals with depression [28, 29]. Copper serves essential roles in mitochondrial respiration, oxidative metabolism, superoxide dismutase activity, and neurotransmitter synthesis [30, 31]. Dysregulation of copper has been associated with oxidative stress, mitochondrial dysfunction, and neuroinflammatory processes in the brain [32, 33, 34]. Our data extend these associations by demonstrating that local copper accumulation within a mood‐relevant cortical region correlates with behavioral indices of depressive‐like states, suggesting that copper imbalance may participate in the manifestation of stress‐related behavioral phenotypes rather than solely reflecting a peripheral epiphenomenon. Although these observations remain correlative, they emphasize the functional significance of copper within specific neural circuits implicated in mood regulation and highlight the prelimbic cortex as a region particularly vulnerable to metal ion dysregulation under conditions of chronic stress. However, the role of aberrant copper accumulation in other brain regions requires further investigation.

Astrocytes are critical regulators of synaptic function and neural homeostasis, and their structural and functional impairments have been repeatedly linked to depressive phenotypes [35, 36, 37]. In the present study, FDX1 expression was preferentially enriched in astrocytes and significantly increased following chronic stress exposure, coinciding with reductions in astrocytic process complexity and calcium signaling capacity. These astrocytic changes were accompanied by deficits in excitatory synaptic transmission and reduced dendritic spine density in adjacent neurons. This pattern is consistent with accumulating evidence that astrocytic morphology and calcium‐dependent signaling are essential for sustaining synaptic plasticity and network stability. Several mechanisms have been proposed to explain how astrocytic changes affect neuronal structure and function. In the prefrontal cortex, astrocytic D1 receptors regulate glutamatergic transmission and synaptic plasticity through D‐serine release, which modulates NMDA receptor function and mEPSC properties [7]. Astrocytic Ca^2+^ signaling also controls the activity of distinct neuronal populations by remodeling proteins at the astrocyte‐neuron interface, including those involved in synaptic signaling and cell adhesion [38]. Moreover, astrocytes limit dendritic activity via an ATP‐adenosine pathway to prevent excessive synaptic depotentiation and maintain spine stability [27]. In our study, stress‐induced reductions in astrocytic Ca^2+^ signaling and process complexity may disrupt these regulatory pathways, leading to impaired excitatory transmission and dendritic spine loss in adjacent neurons.

Mechanistically, increased FDX1 expression may represent a cellular response to local copper accumulation or metabolic stress, which in turn compromises astrocytic physiology and weakens astrocyte‐neuron coupling. Notably, astrocyte‐specific gain‐ and loss‐of‐function manipulations of FDX1 were sufficient to bidirectionally modulate depressive‐like behaviors, supporting an active role for FDX1 beyond that of a passive redox marker. Our data showing increased Lip‐DLAT together with elevated ROS and MDA levels suggest that FDX1 upregulation is associated with mitochondrial metabolic imbalance and oxidative stress in the PrL. Consistent with previous reports that oxidative stress and lipid peroxidation‐related processes can disrupt mitochondrial function and contribute to astrocytic injury, these alterations may participate in the reduced structural complexity of astrocytes observed in our model [39]. However, although our findings implicate Lip‐DLAT and metabolic stress in FDX1‐associated astrocytic dysfunction, direct manipulation of DLAT lipoylation will be required to determine whether this pathway is necessary for astrocytic and behavioral phenotypes. Functionally, such oxidative and metabolic stress may impair astrocytic calcium signaling and the regulation of excitatory synaptic transmission, thereby weakening astrocyte–neuron coupling under depressive conditions.

Physical exercise is widely recognized as an effective non‐pharmacological intervention for depression [40, 41]. In our experiments, exercise normalized copper levels in the prelimbic cortex and reduced astrocytic FDX1 expression, accompanied by improvements in astrocytic calcium dynamics and restoration of neuronal synaptic function. These findings suggest that exercise preserves glial‐neuronal functional coupling under stress conditions, thereby contributing to its behavioral benefits. Although exercise engages multiple molecular and cellular pathways, including neurotrophic signaling, metabolic adaptations, and regulation of oxidative stress [42, 43, 44], our findings identify copper–FDX1 signaling as a region‐ and cell‐type‐specific component that complements existing models of exercise‐induced antidepressant effects.

Although exercise reduced both copper levels and FDX1 expression under CUMS conditions, the observation that FDX1 overexpression‐induced depressive‐like behaviors remained responsive to exercise suggests that exercise may also engage protective mechanisms beyond copper normalization. In this context, our data showing increased expression of SIRT1 and PGC‐1α in the PrL suggests a possible association between exercise‐induced metabolic adaptation and protection against FDX1‐induced dysfunction. Given that PGC‐1α is a key regulator of cellular energy metabolism and has been implicated in neuroplasticity and mitochondrial adaptation, its upregulation may contribute to metabolic resilience and redox balance in the PrL [45]. These effects could act in parallel with copper regulation to buffer the detrimental consequences of FDX1 overexpression and support the restoration of astrocyte–neuron functional coupling.

Several limitations of the present study should be considered. First, although our investigation focused on the prelimbic cortex, whether similar copper‐related astrocytic alterations occur in other depression‐relevant brain regions remains to be determined. Second, interpretation of systemic copper measurements requires caution, as circulating metal ion levels are influenced by age, sex, diet, and comorbid conditions. Validation of our findings in larger and clinically well‐characterized cohorts, with more refined symptom stratification, will therefore be necessary. In addition, our animal experiments were conducted exclusively in male mice; given the well‐established sex differences in depression, future studies incorporating female mice will be important to assess the generalizability of these findings. Third, although our results support a causal role for astrocytic FDX1, future studies employing temporally precise and cell type‐specific manipulations will be essential to clarify the mechanistic hierarchy governing these processes within neural circuits.

## 4 Conclusion

Taken together, our findings demonstrate that copper dyshomeostasis is associated with structural and functional alterations in astrocytes within the prelimbic cortex and identify astrocytic FDX1 as an important component linking metabolic disturbance to synaptic dysfunction and behavioral changes. These results provide new insight into glia‐associated mechanisms in depression and suggest that astrocytes may represent a relevant cellular substrate through which systemic metabolic alterations influence neural circuit function.

## 5 Methods

### 5.1 Human Participants

Twenty participants (10 MDD patients and 10 healthy controls) were assessed using the HAMD‐17. Blood samples were collected for serum copper measurements in all participants. The MDD group additionally underwent a 1‐month standardized aerobic exercise intervention, followed by post‐intervention blood sampling.

Prior to enrollment, all participants underwent physical examination, exercise electrocardiography (ECG), routine blood testing, and vital sign assessment to ensure exercise safety. Exclusion criteria included a history of drug or substance abuse, severe systemic or neurological disorders, and medical conditions that could potentially affect copper metabolism, inflammatory status, or exercise safety. Participants had not received anxiolytics, antidepressants, mood stabilizers, or antipsychotic medications within 1 month prior to enrollment, and had not used medications known to affect copper metabolism within 3 months prior to participation. No pharmacological or psychological treatments were administered during the intervention period.

To standardize the exercise protocol, participants in the exercise group completed moderate‐intensity running training for 1 month (30 min/session, 4 sessions/week). Exercise intensity was maintained at 50–70% of age‐predicted maximal heart rate (calculated as 220 − age). Training sessions were supervised by trained exercise staff, and heart rate was monitored using wearable devices before and after each session to ensure adherence to the target intensity range. Exercise intensity was adjusted when necessary based on monitoring results.

Post‐intervention blood samples were collected at least 24 h after the final exercise session to minimize the acute effects of exercise on circulating copper levels. Participants were monitored regularly throughout the intervention period to ensure safety and adherence. Detailed participant characteristics are provided in Table S1. Ethical approval was obtained from the Ganzhou Hospital Ethics Committee (TY‐HKY2023‐018‐02), and all participants provided written informed consent prior to enrollment.

### 5.2 Copper Measurement

Copper concentrations in serum and tissue samples were quantified using inductively coupled plasma–mass spectrometry (ICP‐MS). Human venous blood and murine whole blood or brain tissues were collected. Serum was separated after clotting for 1 h at room temperature and centrifugation. Samples were digested with concentrated HNO_3_ in sealed PTFE vessels, evaporated to dryness, and reconstituted in 1% HNO_3_ (ultrapure water) prior to ICP‐MS analysis.

### 5.3 Animals

Male C57BL/6J mice (GemPharmatech, Nanjing, China) were housed in a specific pathogen‐free facility at 22°C, 60% humidity, under a 12‐h light/dark cycle. All animal experiments were approved by the Institutional Animal Care and Use Committee of Shanghai Mental Health Center (AWEC‐KY‐2024‐45).

### 5.4 CUMS and Exercise Intervention

Except for controls, mice underwent a 4‐week CUMS protocol, which included multiple stressors applied in a semi‐random order (1–2 per day, no consecutive repetition). Daily stressors included tail pinch, restraint, foreign object exposure, cold water swimming, cage shaking, wet bedding, water/food deprivation, strobe light, reversed light cycle, cage tilt, and odor exposure. Post‐CUMS, the exercise group performed daily treadmill training at 10 m/min for 1 h/day.

### 5.5 Behavioral Assessments

#### 5.5.1 Open Field Test

After 30‐min acclimation, mice were placed in the center of a 40×40×40 cm open field for a 10‐min test. Behavior was recorded and analyzed using EthoVision XT (Noldus). Time in the center zone and total distance traveled were measured. The arena was cleaned with 75% ethanol between trials.

#### 5.5.2 Forced Swim Test

Mice were placed in a transparent cylinder (30 cm height × 20 cm water depth) at 22°C for 6 min. Immobility time during the last 5 min was recorded as a measure of behavioral despair.

#### 5.5.3 Tail Suspension Test

Mice were suspended individually by the tail 30 cm above a surface for 6 min. Immobility time during the last 5 min was quantified as an index of despair‐like behavior.

#### 5.5.4 Sucrose Preference Test

After single housing and 24‐h adaptation to two bottles of 1.5% sucrose solution, one bottle was replaced with water. Bottle positions were switched every 12 h. Following 24 h of food/water deprivation, mice were given access to both fluids for 24 h. Sucrose preference was calculated as (sucrose intake / total fluid intake) × 100%.

#### 5.5.5 Copper‐Enriched Diet Administration

The copper‐enriched diet (catalog no. SYHCu4000; copper content: 4000 ppm) was obtained from Anhui Kuibu Shuyu Biotechnology Co., Ltd. Compared with standard chow, the copper‐enriched diet was supplemented with copper carbonate (CuCO_3_) to achieve the indicated copper concentration. Mice in the experimental group were allowed ad libitum access to the copper‐enriched diet for two consecutive weeks, whereas control mice received standard chow throughout the experimental period.

#### 5.5.6 TTM Treatment

Ammonium tetrathiomolybdate (TTM; MedChemExpress) was initially dissolved in DMSO to prepare a stock solution and subsequently diluted with sterile saline to a final concentration of 4 g/L. One week before behavioral testing, mice in the control and CUMS groups received the vehicle (saline, 0.2 mL per mouse) by oral gavage once daily. Mice in the TTM‐treated group were administered TTM by oral gavage at a dose of 30 mg/kg (0.2 mL per mouse) once daily.

#### 5.5.7 Immunofluorescence

Mice were transcardially perfused with PBS, followed by brain fixation in 4% PFA and cryoprotection in 30% sucrose. After OCT embedding, 30‐µm coronal sections were prepared using a cryostat. Following blocking with 5% BSA, sections were incubated with primary antibodies (c‐Fos, S100β, FDX1, NeuN, and Olig2, all from Abcam) and corresponding Alexa Fluor‐conjugated secondary antibodies (Thermo Fisher). Immunofluorescence imaging was performed using a confocal microscope after sections were mounted with DAPI.

#### 5.5.8 RNA Extraction and qPCR

Total RNA was isolated using TRIzol reagent (Invitrogen) and reverse transcribed into cDNA using the PrimeScript RT kit (TAKARA). Quantitative PCR was conducted with TB Green Premix Ex Taq II (TAKARA) on a QuantStudio 6 Flex system. All reactions were performed in triplicate. Relative gene expression was determined via the 2^−ΔΔCt^ method, with GAPDH serving as the internal control. Primer sequences are provided in Table S2.

#### 5.5.9 Detection of ROS

Intracellular ROS levels were assessed using two complementary methods: DCFH‐DA staining followed by flow cytometric analysis of single‐cell suspensions prepared from brain tissue, and DHE staining in PrL cortical sections. DCF fluorescence was quantified by flow cytometry, while DHE fluorescence intensity in the PrL was analyzed using fluorescence microscopy.

#### 5.5.10 Measurement of Lipid Peroxidation by MDA Assay

Lipid peroxidation was assessed by measuring MDA with a commercial assay kit (Beyotime). Procedures followed the manufacturer's protocol. Absorbance at 532 nm was measured, and MDA levels were calculated from a standard curve.

#### 5.5.11 Western Blot

Tissue samples were homogenized in RIPA lysis buffer containing protease and phosphatase inhibitors. Protein concentration was determined by BCA assay, and equal amounts (20 µg) were separated by SDS‐PAGE, transferred to PVDF membranes, and blocked with 5% BSA. Membranes were incubated overnight at 4°C with primary antibodies against FDX1 (HUABIO), Lip‐DLAT (Abcam), DLAT (Abcam), PGC‐1α (Abcam), SIRT1 (HUABIO), and GAPDH (HUABIO), followed by secondary antibody incubation. Signals were detected using a chemiluminescent substrate and quantified in ImageJ, with normalization to GAPDH.

#### 5.5.12 Astrocyte Morphology Analysis

PrL astrocytes were immunostained with anti‐S100β antibodies. Z‐stack images were captured on an Olympus FV1200 confocal microscope (63× objective) and processed into maximum intensity projections in ImageJ. Astrocyte morphology and Sholl analysis were conducted using ImageJ software.

#### 5.5.13 Primary Astrocyte Culture and Copper Treatment

Primary astrocytes were isolated from the cerebral cortices of postnatal day 3 C57BL/6 mice and cultured in DMEM (Gibco) supplemented with 10% fetal bovine serum at 37°C in a humidified incubator containing 5% CO_2_. After 7 days in vitro, microglia and other contaminating cells were removed by orbital shaking at 240 rpm for 3–4 h. The remaining astrocytes were subsequently replated onto glass‐bottom confocal dishes for further experiments.

To induce intracellular copper accumulation, astrocytes were treated with CuCl_2_ (Sigma–Aldrich) together with elesclomol (ES; MedChemExpress) for 2 h. Control groups received either vehicle alone or an equal volume of ES‐containing vehicle. Following treatment, cells were washed with phosphate‐buffered saline (PBS) and incubated with 0.8 µm Copper Fluor‐4 (CF4; MedChemExpress) probe at 37°C for 10 min in the dark. After removal of the unbound probe by PBS washing, cells were further incubated for an additional 20 min. Fluorescence images were then acquired using an Olympus confocal microscope under identical imaging settings across all groups. Fluorescence intensity was quantified from randomly selected fields using ImageJ software, and data were averaged from at least three independent experiments.

#### 5.5.14 Stereotaxic Virus Injection

Mice were anesthetized, and the skull was exposed and secured in a stereotaxic apparatus (RWD). Coordinates for the PrL were determined relative to Bregma: anterior‐posterior 1.87 mm, mediolateral 0.30 mm, dorsoventral −2.10 mm. A small cranial hole was drilled, and a glass micropipette was used to inject the virus at 0.1 µL/min. The pipette was left in place for 10 min post‐injection before slow withdrawal. The incision was sutured, and mice were allowed to recover on a heating pad. Viruses used in this study include: rAAV‐GfaABC1D‐mCherry‐5'‐miR‐30a‐shRNA(Fdx1)‐3'‐miR‐30a‐WPRE, rAAV‐GfaABC1D‐mCherry‐5'‐miR‐30a‐shRNA(scramble)‐3'‐miR‐30a‐WPRE, rAAV‐GfaABC1D‐Fdx1‐P2A‐mCherry‐WPRE, rAAV‐GfaABC1D‐mCherry‐WPRE, rAAV‐hSyn‐mCherry‐5'‐miR‐30a‐shRNA(Fdx1)‐3'‐miR‐30a‐WPRE, rAAV‐hSyn‐mCherry‐5'‐miR‐30a‐shRNA(scramble)‐3'‐miR‐30a‐WPRE.

#### 5.5.15 In Vivo Two‐Photon Calcium Imaging

Mice received stereotaxic injections of a GCaMP6f‐expressing viral vector (rAAV2/5‐GfaABC1D‐cyto‐GCaMP6f‐WPRE) into the PrL and were implanted with an imaging window. After one week of recovery and habituation, calcium activity was recorded using a two‐photon microscope. Calcium activity was expressed as ΔF/F_0_.

### 5.6 Whole‐Cell Patch‐Clamp Recording

#### 5.6.1 Acute Brain Slice Preparation

Anesthetized mice were perfused with ice‐cold, oxygenated sucrose‐ACSF. Coronal PrL slices (280 µm) were prepared using a vibratome, recovered at 34°C for 30 min, and then held at room temperature. Recording ACSF contained (in mm): 124 NaCl, 2.5 KCl, 2 MgSO_4_, 2.5 CaCl_2_, 1.25 NaH_2_PO_4_, 22 NaHCO_3_, and 10 glucose.

#### 5.6.2 Electrophysiological Recording

Brain slices were perfused with ACSF (29°C–31°C). Neurons were visualized via IR‐DIC optics and recorded using borosilicate pipettes (3–5 MΩ, K‐gluconate‐based internal solution). mEPSCs were recorded at −70 mV with TTX, CGP52432, and picrotoxin. Action potentials were evoked in current‐clamp using depolarizing steps (0–250 pA) in the presence of CNQX and picrotoxin. Signals were amplified (Multiclamp 700B), filtered at 3 kHz, digitized at 10 kHz (Digidata 1440A), and analyzed offline with Clampfit and Origin.

#### 5.6.3 Golgi Staining

PrL blocks (∼5 mm) were Golgi‐stained (GENMED rapid kit). After 24 h fixation, tissues were impregnated in the dark for 2 weeks, cryoprotected (30% sucrose), sectioned at 40 µm (Leica CM1950), and processed per kit instructions. Sections were developed, dehydrated, cleared, and mounted in neutral mounting medium for imaging and analysis.

#### 5.6.4 Single‐Cell RNA Sequencing

Single‐cell RNA sequencing libraries were prepared using the 10x Genomics Chromium or DNBelab C platform. Processed cells were cryopreserved and shipped for library construction. Libraries (10–100 ng RNA, DV200 ≥ 30%) were sequenced on the DNBSEQ‐G400 or DNBSEQ‐T7 platform. Downstream analyses, including cell‐type identification and cellular heterogeneity analysis, were performed using standard bioinformatics pipelines.

#### 5.6.5 Statistical Analysis

Data are presented as mean ± SEM. Normality was assessed using the Shapiro–Wilk test. Parametric data were analyzed using unpaired two‐tailed Student's *t*‐tests or one‐way ANOVA followed by Tukey's post hoc test, as appropriate. Non‐parametric data were analyzed using the Mann–Whitney U test or Kruskal–Wallis test. Statistical significance was defined as p < 0.05. Data analysis and graphing were performed using GraphPad Prism version 10.1.0.

## Author Contributions

L.G. and R.H. contributed equally to this work, performed the majority of the experiments, and drafted the manuscript. Z.T., T.F., Z.H., X.Z., and P.J. carried out key experiments and data acquisition. H.Z. participated in data analysis and provided technical support. K.‐F.S., T.L., Y.C., and L.Y. conceived and supervised the study and revised the manuscript critically. L.Y., Y.C., and H.Z. secured funding support. All authors reviewed and approved the final manuscript.

## Ethical Statement

The human study was approved by the Ganzhou Hospital Ethics Committee (TY‐HKY2023‐018‐02), and all participants signed informed consent forms prior to inclusion in the study. All animal experiments were conducted in accordance with the guidelines outlined in the National Institutes of Health Guide for the Care and Use of Laboratory Animals and were approved by the Institutional Animal Care and Use Committee of Shanghai Mental Health Center (AWEC‐KY‐2024‐45).

## Conflicts of Interest

The authors declare no conflicts of interest.

## Supporting information




**Supporting File**: advs76088‐sup‐0001‐SuppMat.docx.

## Data Availability

The data that support the findings of this study are available from the corresponding author upon reasonable request.
