# Supplementary material for: Astrocytic FDX1 Contributes to Copper Dyshomeostasis‐associated Synaptic Dysfunction in Depression and Is Modulated by Exercise
Source: Adv Sci (Weinh). 2026 Jun 15:e76088. Online ahead of print. doi: 10.1002/advs.76088 (PMC13336564; doi:10.1002/advs.76088)
Supplement: Supplementary file 1 — Supporting File: advs76088‐sup‐0001‐SuppMat.docx. [file ADVS-9999-e76088-s001.docx]

**Astrocytic FDX1 contributes to copper dyshomeostasis-associated synaptic dysfunction in depression and is modulated by exercise**

*Lina Gao ^†^, Rongji Hui ^†^, Zhibo Tang, Tao Feng, Zhihang Hu, Xiaoqing Zhang, Ping Jiang, Hui Zhao, Kwok-Fai So *, Tianyuan Luo *, Yanzhou Chang *, Lan Yan **

Zhibo Tang, Xiaoqing Zhang, Ping Jiang, Lan Yan

Shanghai Mental Health Center, School of Medicine, Shanghai Jiao Tong University, Shanghai 200030, China

E-mail: Lan Yan, 13247672725@163.com

Lina Gao

School of Mental Health, Wenzhou Medical University, Wenzhou 325035, China

Rongji Hui, Tao Feng

College of Forensic Medicine, Hebei Medical University, Hebei Key Laboratory of Forensic Medicine, Collaborative Innovation Center of Forensic Medical Molecular Identification, Hebei Province, Shijiazhuang 050017, China

Rongji Hui

Hebei Medical University Basic Medicine Postdoctoral Research Station, Hebei Province, Shijiazhuang 050017, China

Zhihang Hu

Liver Transplantation Center, Department of General Surgery, Huashan Hospital, Fudan University, Shanghai 200040, China

Hui Zhao

Department of Psychiatry, Ganzhou Hospital-Nanfang Hospital, Southern Medical University (Ganzhou People's Hospital), Ganzhou, Jiangxi Province, China

Yanzhou Chang

College of Chemistry and Materials Science, Jinan University, Guangzhou 510632, China

E-mail: Yanzhou Chang, 377966911@qq.com

Tianyuan Luo

Department of Anesthesiology, Affiliated Hospital of Zunyi Medical University, Guizhou Province, Zunyi 563100, China

Key Laboratory of Anesthesia and Organ Protection of Ministry of Education (In Cultivation), Zunyi Medical University, Guizhou Province, Zunyi 563100, China

E-mail: Tianyuan Luo, 407542949@qq.com

Kwok-Fai So

GHM Institute of CNS Regeneration, Jinan University, Guangzhou 510600, China

E-mail: Kwok-Fai So, hrmaskf@hku.hk

**Keywords:** Depression, Astrocytes, Copper metabolism, Prelimbic cortex, Exercise


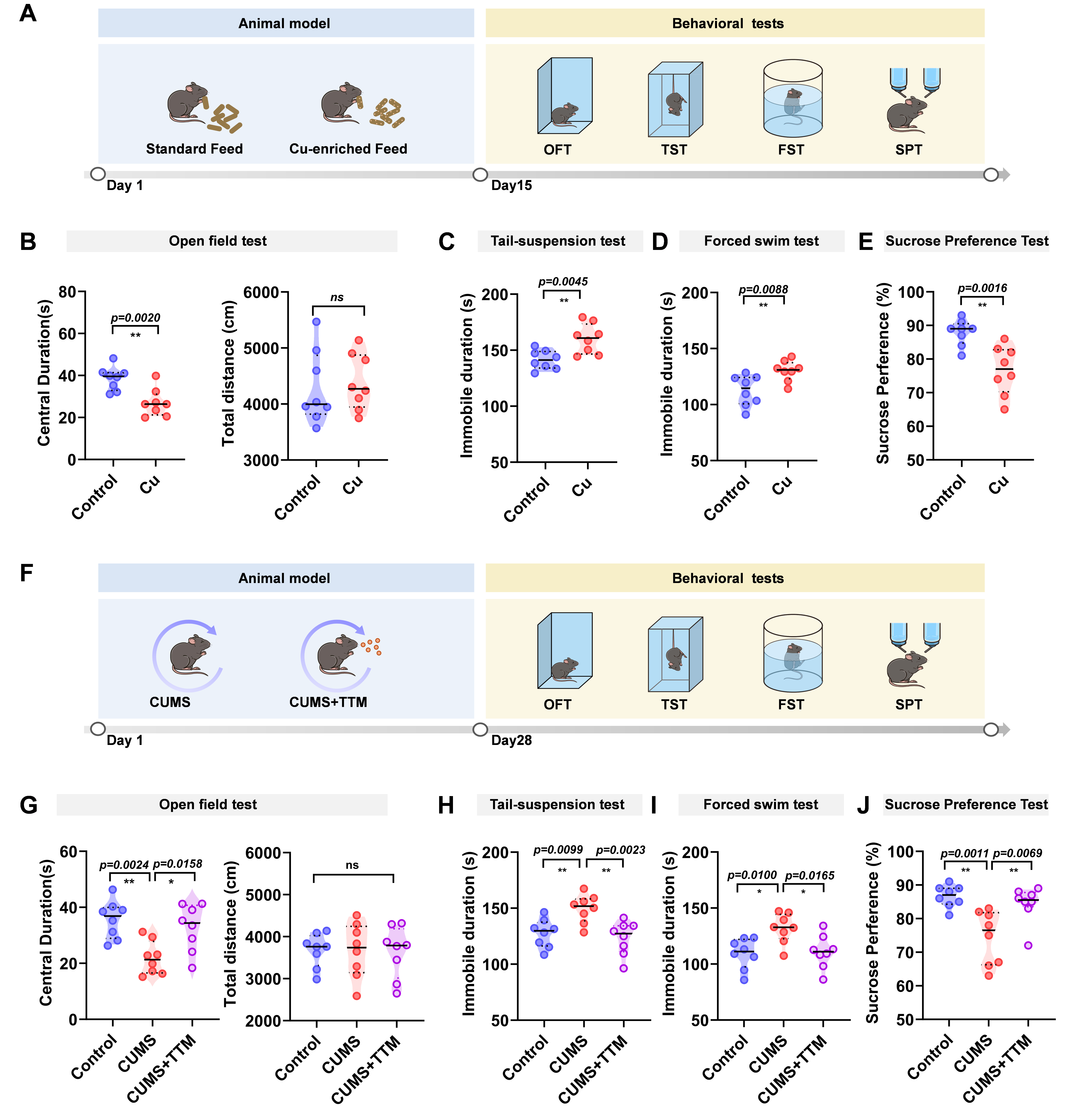


**Supplementary Figure 1. Copper concentration influences depressive-like behaviors in mice.** (A) Experimental timeline of a high-copper diet–induced mouse model. (B) Open field test: center time (unpaired two tailed t test, t(14) = 3.793, P = 0.0020; left) and total distance (t(14) = 0.3470, P = 0.7337; right). n = 8/group. (C) Tail suspension test: immobility time (t(14) = 3.376, P = 0.0045; n = 8/group). (D) Forced swim test: immobility time (t(14) = 3.041, P = 0.0088; n = 8/group). (E) Sucrose preference index (t(14) = 3.888, P = 0.0016; n = 8/group).(F) Experimental timeline of the chronic unpredictable mild stress (CUMS) model with or without TTM treatment. (G) Open field test: center time (one-way ANOVA with post hoc comparisons, Control vs CUMS, p = 0.0024; CUMS vs CUMS+TTM, p = 0.0158; left) and total distance (ns; right). n = 8/group. (H) Tail suspension test: immobility time (Control vs CUMS, p = 0.0099; CUMS vs CUMS+TTM, p = 0.0023; n = 8/group). (I) Forced swim test: immobility time (Control vs CUMS, p = 0.0100; CUMS vs CUMS+TTM, p = 0.0165; n = 8/group). (J) Sucrose preference test: sucrose preference index (Control vs CUMS, p = 0.0011; CUMS vs CUMS+TTM, p = 0.0069; n = 8/group).


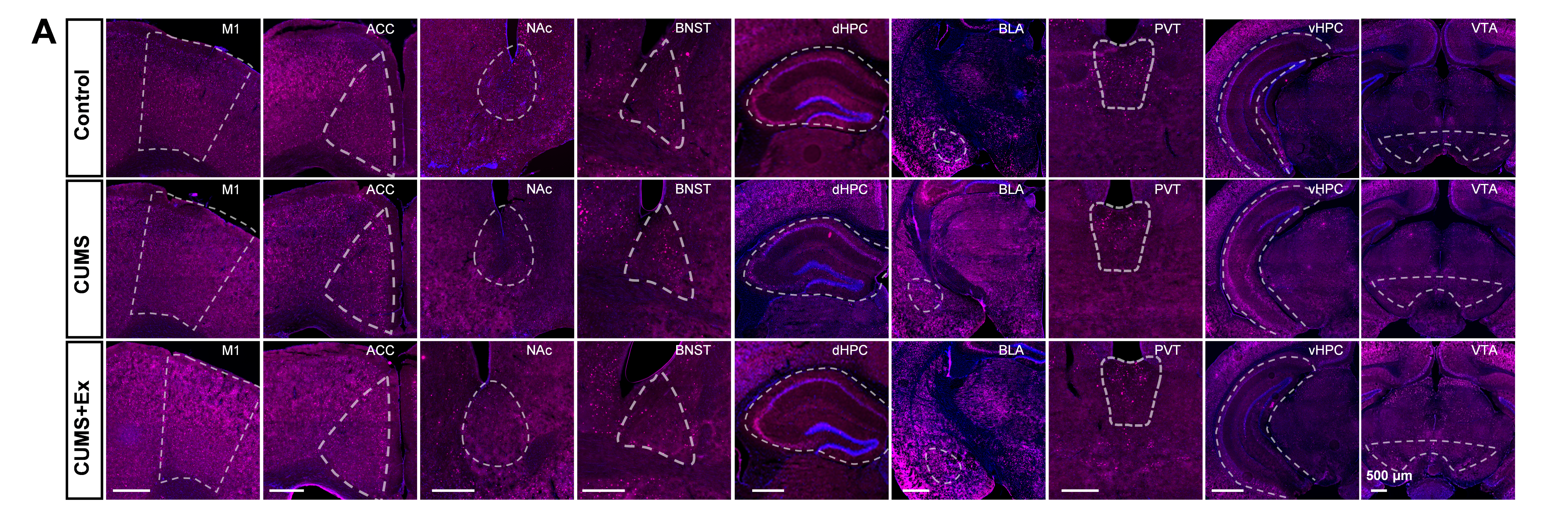


**Supplementary Figure 2. Representative immunofluorescence images of c-Fos.** (A) Representative schematic of c-Fos staining in additional brain regions.


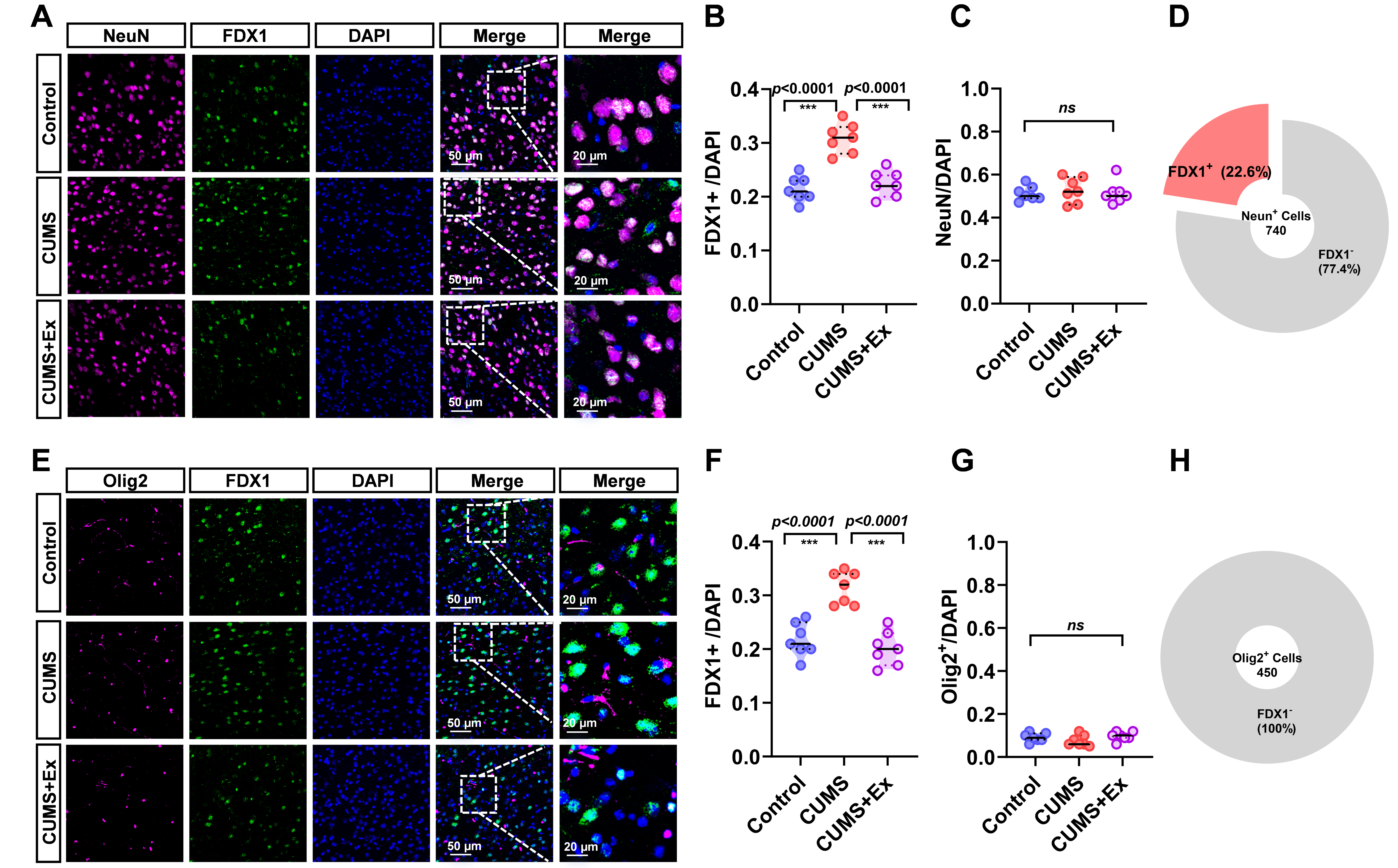


**Supplementary Figure 3. Co-localization of FDX1 with neurons and oligodendrocytes in the PrL.** (A) Representative immunofluorescence images of FDX1 co‑localization with NeuN‑positive neurons in PrL. (B, C) Exercise reversed the CUMS‑induced increase in FDX1‑positive cells but did not alter NeuN‑positive cell proportions (one‑way ANOVA: B, F (2, 18) = 29.06, P < 0.0001; C, F(2,18) = 0.2006, P = 0.8200; n = 7/group). (D) Proportion of FDX1‑positive cells among NeuN‑positive neurons. (E) Representative immunofluorescence images of FDX1 co‑localization with oligodendrocyte marker Olig2 in PrL. (F, G) Exercise reversed the CUMS‑induced increase in FDX1‑positive cells without affecting Olig2‑positive cell proportions (one‑way ANOVA: F, F (2, 18) = 26.75, P < 0.0001; G, F(2,18) = 1.724, P = 0.2066; n = 7/group). (H) Proportion of FDX1‑positive cells among Olig‑positive cells.


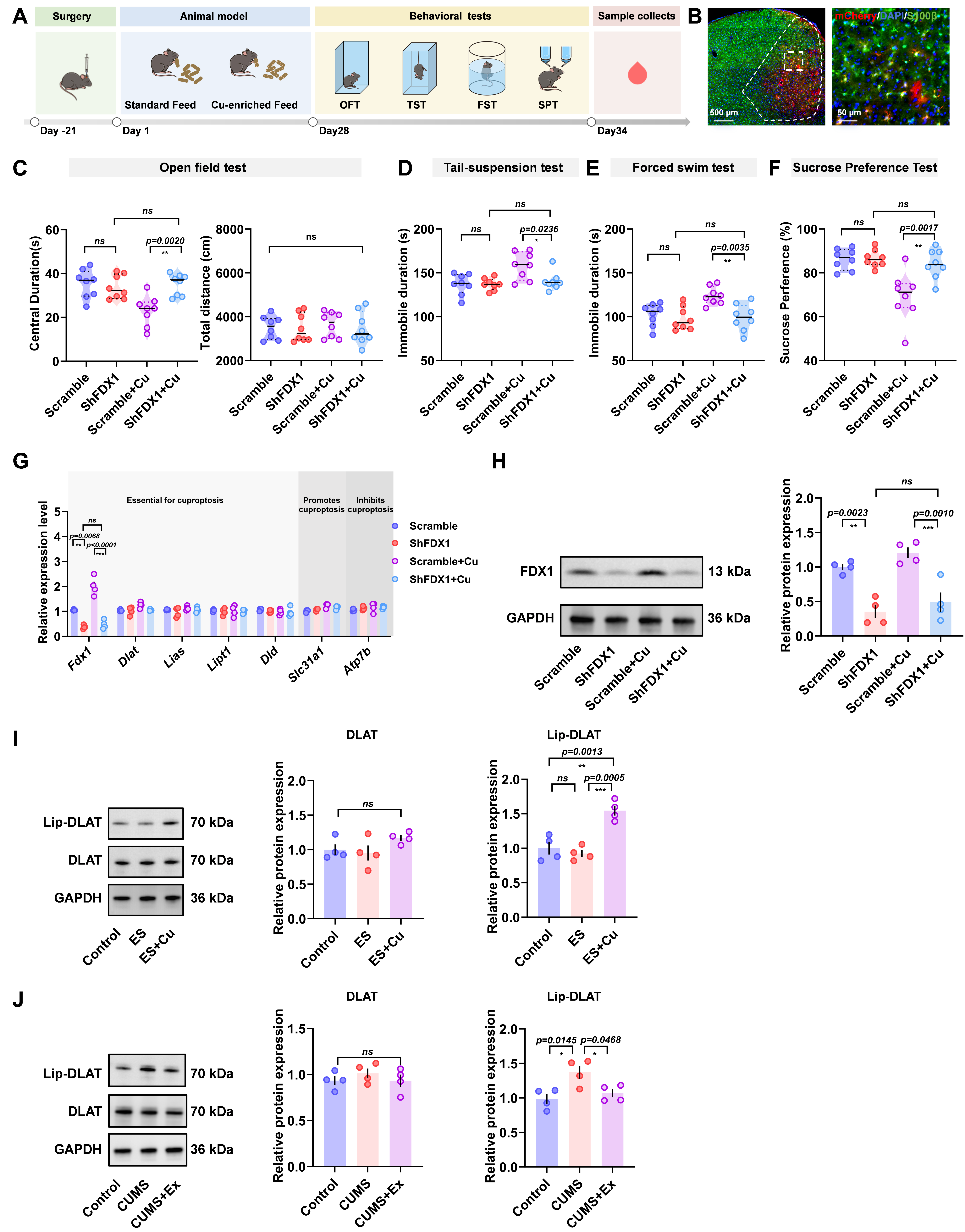
**Supplementary Figure 4. Astrocytic FDX1 knockdown alleviates copper-induced depressive-like behaviors.**

(A) Experimental timeline showing stereotaxic viral injection, copper-enriched diet exposure. (B) Representative immunofluorescence images showing viral injection sites. (C–F) Astrocytic FDX1 knockdown attenuated copper-induced depressive-like behaviors: open field center duration: F (3, 28) = 7.902, P=0.0006; total distance: F (3, 28) = 0.1260, P=0.9439. Tail suspension: F (3, 28) = 6.127, P=0.0024. Forced swim: F (3, 28) = 7.102, P=0.0011. Sucrose preference: F (3, 28) = 10.45, P<0.0001. n = 8/group. (G) Relative mRNA expression of copper metabolism-related genes (two-way ANOVA, F (18, 84) = 17.87, P < 0.0001, n = 4/group). (H) Western blot analysis of FDX1 protein in PrL and quantification (one-way ANOVA, F (3, 12) = 17.78, P=0.0001, n = 4/group). (I) Representative western blot images and quantification of DLAT (one-way ANOVA, F (2, 9) = 2.036, P=0.1864; middle) and Lip-DLAT (one-way ANOVA, F (2, 9) = 21.79, P=0.0004; right) protein expression in primary astrocytes. n = 4/group. (J) Representative western blot images and quantification of DLAT (one-way ANOVA, F (2, 9) = 0.6614, P=0.5395; middle) and Lip-DLAT (one-way ANOVA, F (2, 9) = 7.171, P=0.0137; right) protein expression in the PrL. n = 4/group.


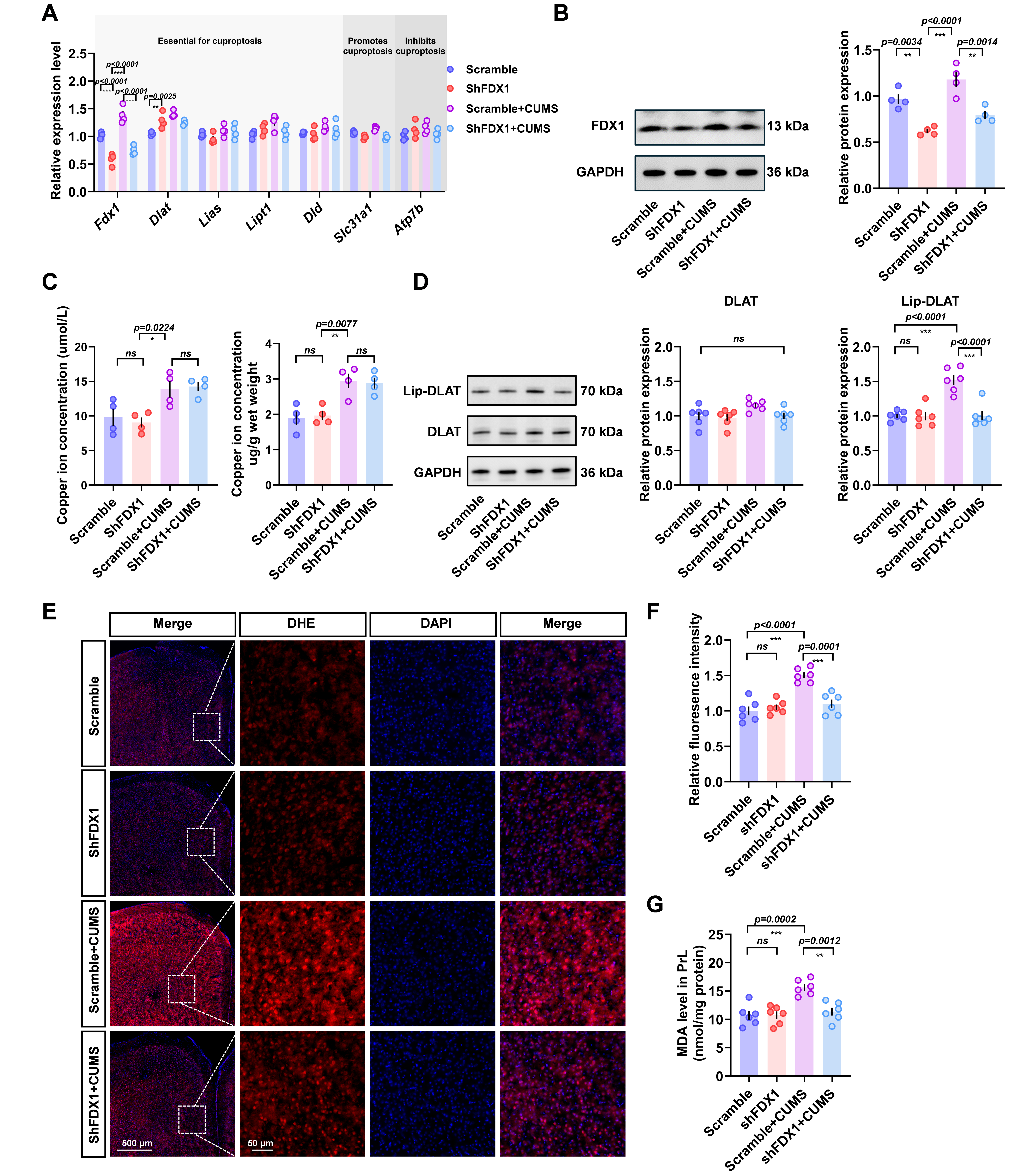


**Supplementary Figure 5. Effects of astrocytic FDX1 knockdown on copper metabolism, Lip-DLAT, and oxidative stress.** (A) Relative mRNA expression of copper metabolism-related genes (two-way ANOVA, F (18,84) = 7.832, P<0.0001, n=4/group). (B) Western blot analysis of FDX1 protein in PrL and quantification (one-way ANOVA, F (3,12) = 19.55, P<0.0001, n=4/group). (C) Copper concentrations in serum (one-way ANOVA, F (3, 12) = 7.413, P=0.0045 left) and Copper concentrations in PrL (F (3, 12) = 11.06, P=0.0009; right). n = 4/group. (D) Representative western blot images and quantification of DLAT (one-way ANOVA, F (3, 20) = 2.963, P=0.0568; middle) and Lip-DLAT (one-way ANOVA, F (3, 20) = 19.43, P<0.0001; right) protein expression in the PrL. n = 6/group. (E, F) Representative DHE staining in PrL (E) and quantified fluorescence (F) (one‑way ANOVA, F (3, 20) = 19.63, P < 0.0001; n = 6/group). (G) MDA levels in PrL (one‑way ANOVA, F (3, 20) = 12.46, P<0.0001; n = 6/group).


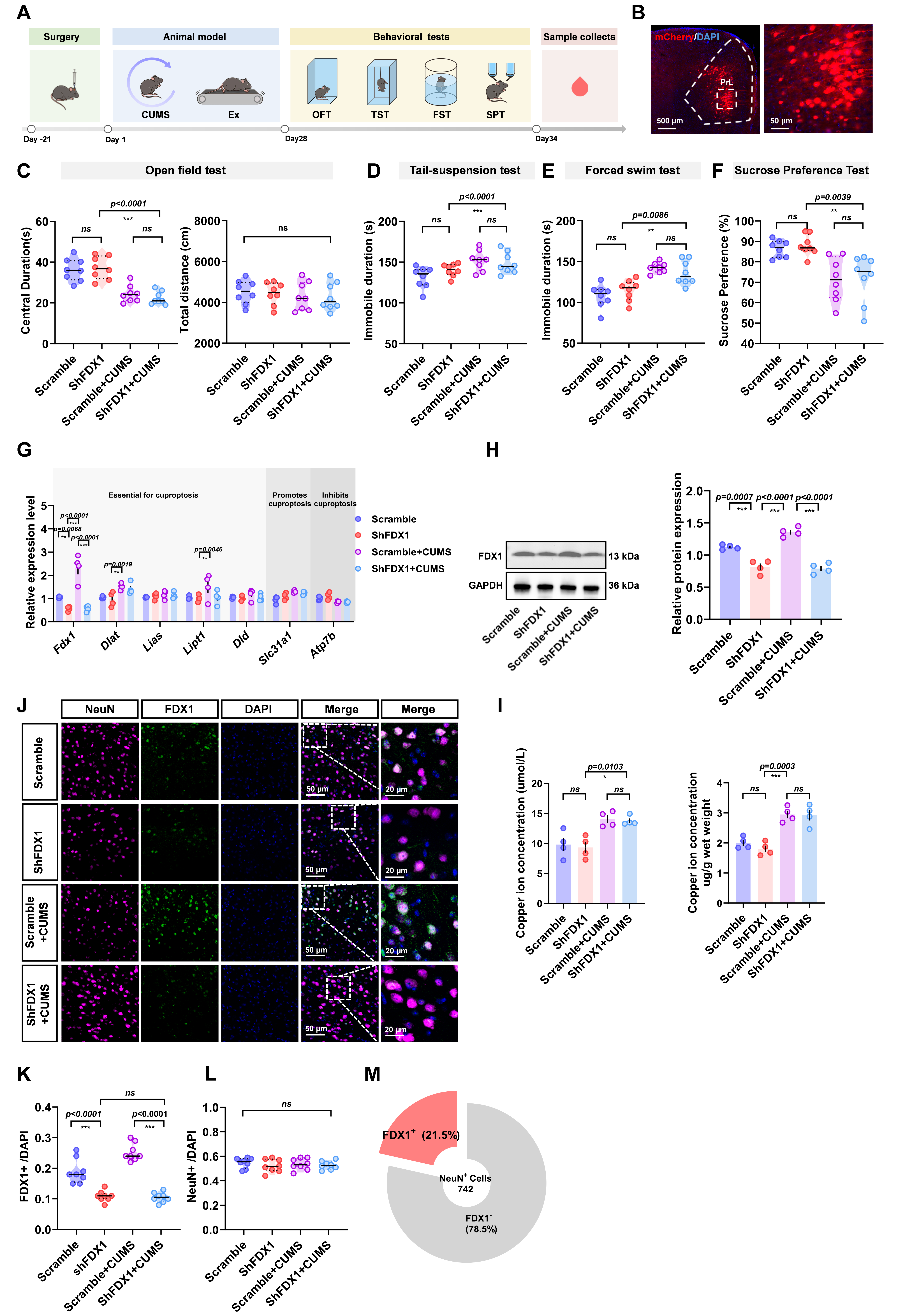


**Supplementary Figure 6. Neuronal knockdown of FDX1 in the PrL does not affect CUMS-induced depressive-like behaviors.** (A) Experimental timeline. Mice received stereotaxic injection of rAAV-hSyn-mCherry-5'-miR-30a-shRNA(Fdx1)-3'-miR-30a-WPRE or control virus rAAV-hSyn-mCherry-5'-miR-30a-shRNA(scramble)-3'-miR-30a-WPRE. (B) Viral injection sites in PrL. (C–F) Neuronal FDX1 knockdown did not alleviate CUMS‑induced depressive‑like behaviors: open‑field center time (F(3,28) = 21.76, P < 0.0001), distance (F(3,28) = 0.2362, P = 0.8703); tail suspension (F(3,28) = 4.814, P = 0.0079); forced swim (F(3,28) = 14.26, P < 0.0001); sucrose preference (F(3,28) = 9.003, P = 0.0002). n = 8/group. (G) Copper metabolism gene mRNA levels (two‑way ANOVA, F(18,84) = 9.431, P < 0.0001; n = 4/group). (H) FDX1 protein in PrL (one‑way ANOVA, F(3,12) = 47.16, P < 0.0001; n = 4/group). (I) Copper concentrations in serum (one-way ANOVA, F (3, 12) =9.616, P=0.0016 left) and Copper concentrations in PrL(F (3, 12) =21.37, P< 0.0001; right). n = 4/group. (J) FDX1 co‑localization with NeuN‑positive neurons in PrL. (K, L) FDX1‑positive cells (F (3, 28) = 53.90, P < 0.0001) and NeuN‑positive cells (F(3,28) = 0.4513, P = 0.7184). n = 4/group. (M) Proportion of FDX1‑positive cells among NeuN‑positive neurons.


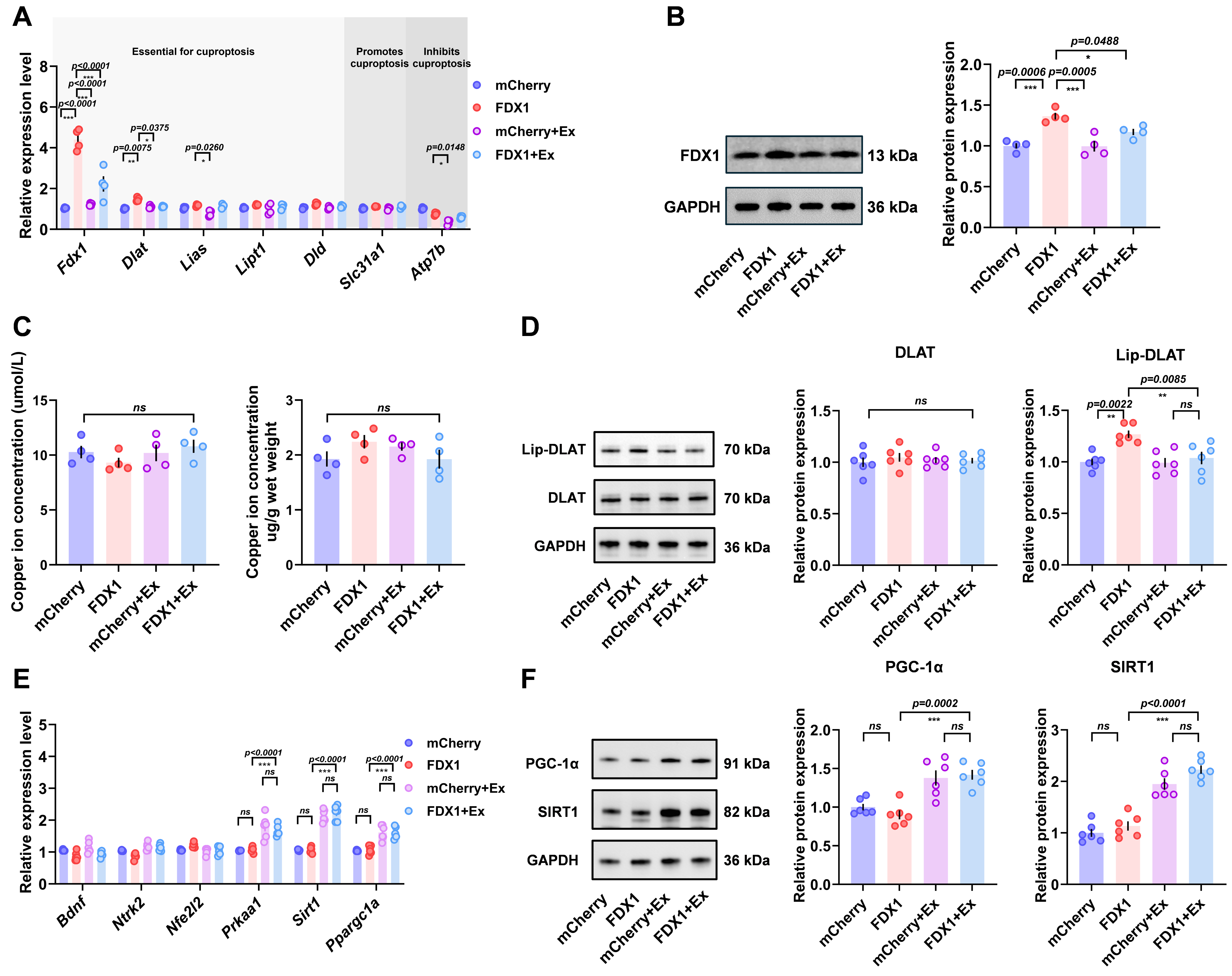


**Supplementary Figure 7. Overexpression of FDX1 does not affect copper levels.** (A) Relative mRNA expression of copper metabolism‑related genes (two‑way ANOVA, F(18,84) = 34.12, P < 0.0001; n = 4/group). (B) Western blot of FDX1 protein in PrL and quantification (one‑way ANOVA, F(3,12) = 14.48, P = 0.0003; n = 4/group). (C) Copper concentrations in serum (one-way ANOVA, F (3, 12) =1.064, P=0.4008 left) and Copper concentrations in PrL(F (3, 12) = 1. 493, P=0.2664; right). n = 4/group. (D) Representative western blot images and quantification of DLAT (one-way ANOVA, F (3, 20) = 0.2831, P=0.8370; middle) and Lip-DLAT (one-way ANOVA, F (3, 20) = 8.410, P=0.0008; right) protein expression in the PrL. n = 6/group. (E) Relative mRNA expression of Bdnf, Ntrk2, Nfe2l2, Prkaa1, Sirt1, and Ppargc1a in the PrL (two-way ANOVA, F (15, 120) = 21.19, P < 0.0001, n = 6/group). (F) Western blot analysis of PGC-1α (one-way ANOVA, F (3, 20) = 14.71, P<0.0001; middle) and SIRT1(one-way ANOVA, F (3, 20) = 44.16, P<0.0001; right) protein expression in the PrL and quantification. n = 6/group.

**Supporting Table 1.** Baseline characteristics of healthy controls and patients with MDD.

|  | Healthy control group(n=10) | MDD patient group(n=10) |
| --- | --- | --- |
| Age (years) | 27.10 ± 3.45 | 28.00 ± 5.47 |
| Sex | 5 male / 5 female | 5 male / 5 female |
| HAMD score (baseline) | 3.30 ± 1.33 | 22.30 ± 3.97 |
| HAMD score (after exercise) | / | 7.90 ± 1.59 |
| BMI (kg/m²) | 21.86 ± 2.17 | 20.97 ± 2.82 |
| Systolic blood pressure (mmHg) | 121.50 ± 10.48 | 118.75 ± 13.26 |
| Diastolic blood pressure (mmHg) | 71.50 ± 6.98 | 69.00 ± 10.27 |
| Resting heart rate (beats/min) | 71.40 ± 6.31 | 72.79 ± 5.27 |
| Antidepressant use, n (%) | 0 (0%) | 0 (0%) |
| Occupational metal exposure, n (%) | 0 (0%) | 0 (0%) |
| High-copper diet (self-reported), n (%) | 0 (0%) | 0 (0%) |
| History of copper metabolism disorders, n (%) | 0 (0%) | 0 (0%) |
| Use of copper-interfering medications, n (%) | 0 (0%) | 0 (0%) |

**Supporting Table 2.** Primer sequences used for qRT-PCR.

| Gene | Forward | Reverse |
| --- | --- | --- |
| ***Fdx1*** | GTTAGATGCCATTACTGATGAAGAG | CTTCAGGCACACGCACAGTCAT |
| ***Dlat*** | CTGAGTGAAGGAGACTTGCTGG | TCCCAGAGGAACATCCCTTGTG |
| ***Lias*** | CACATCGCCAAGACCGTGTCAT | AGACAGAGCCACCTTCTCCACT |
| ***Lipt1*** | CTTCTTCGCTGAAGAGCCCGTA | CTTCACAGGTCAGCGTGGAATC |
| ***Dld*** | GGGTTGGCAAATCGGAAGAACAG | TCACCATGCCATCTGTGTCAGC |
| ***Slc31a1*** | CGCTACAATTCCATGCCTGTCC | GACTACCTGGATGATGTGCAGC |
| ***Atp7b*** | ATCATCCCAGGACTGTCCGTTC | ATGTTGGCGGACCTGTGTCTCA |
| ***Hmox1*** | CACTCTGGAGATGACACCTGAG | GTGTTCCTCTGTCAGCATCACC |
| ***Nos2*** | GAGACAGGGAAGTCTGAAGCAC | CCAGCAGTAGTTGCTCCTCTTC |
| ***Nfe2l2*** | CAGCATAGAGCAGGACATGGAG | GAACAGCGGTAGTATCAGCCAG |
| ***Mt1*** | GGATGGACACAGAGAACTTCGTG | CGAGAGGTAGTTCTGGGTTGAG |
| ***Tpx*** | TGACGTGGATGTGCAGAAGATAG | CAAAGGACGTGGCTGCACATGA |
| ***Gsr*** | TCCAGAGCTTGAAGGTGTTGCC | AACCAAGGGAGCTTCAGGGTCA |
| ***Cat*** | CGGCACATGAATGGCTATGGATC | AAGCCTTCCTGCCTCTCCAACA |
| ***Gsta1*** | TGGAAGGAGGAGGTGGTTACCA | GGTAAAGGGTGAGGTCTCCATC |
| ***Mpo*** | CGTCCAGCTACATCGCTCACTT | CAGGTCATTCTCTGGTTTGCCG |
| ***Sod2*** | TAACGCGCAGATCATGCAGCTG | AGGCTGAAGAGCGACCTGAGTT |
| ***Gpx4*** | CCTCTGCTGCAAGAGCCTCCC | CTTATCCAGGCAGACCATGTGC |
| ***Gpx1*** | AGCAGCTGCTGCACTCTACCAG | TGAACGCCGTCCGCATCCTCT |
| *Bdnf* | GGCTGACACTTTTGAGCACGTC | CTCCAAAGGCACTTGACTGCTG |
| *Ntrk2* | CCACGGATGTTGCTGACCAAAG | GCCAAACTTGGAATGTCTCGCC |
| *Prkaa1* | GGTGTACGGAAGGCAAAATGGC | CAGGATTCTTCCTTCGTACACGC |
| *Sirt1* | GGAGCAGATTAGTAAGCGGCTTG | GTTACTGCCACAGGAACTAGAGG |
| *Ppargc1a* | GAATCAAGCCACTACAGACACCG | CATCCCTCTTGAGCCTTTCGTG |
